# Wild *Asparagus* Shoots Constitute a Healthy Source of Bioactive Compounds

**DOI:** 10.3390/molecules28155786

**Published:** 2023-07-31

**Authors:** Tarik Chileh Chelh, Miguel A. Rincon-Cervera, Francisco Gomez-Mercado, Rosalia Lopez-Ruiz, Manuela Gallon-Bedoya, Mohamed Ezzaitouni, Jose L. Guil-Guerrero

**Affiliations:** 1 Food Technology Division, ceiA3, CIAMBITAL, University of Almeria, 04120 Almeria, Spain; chileh@hotmail.es (T.C.C.); mrc883@ual.es (M.A.R.-C.); mohammedgamn@gmail.com (M.E.); 2 Institute of Nutrition and Food Technology, University of Chile, Macul, Santiago 7830490, Chile; 3 Biology and Geology Department, University of Almeria, 04120 Almeria, Spain; frgomez@ual.es; 4 Chemical-Physical Department, Analytical Chemistry of Pollutants, University of Almeria, 04120 Almeria, Spain; rosalialr@ual.es; 5 Faculty of Agricultural Sciences, Department of Agricultural and Food Engineering, Medellín Campus, National University of Colombia, Medellin 050034, Colombia; mgallonb@unal.edu.co

**Keywords:** *Asparagus*, phenolic compounds, flavonoids, vitamin C, antioxidant activity, MTT, HT-29 cells

## Abstract

Wild *Asparagus* shoots are consumed worldwide, although most species remain understudied. In this work, a total of four wild *Asparagus* species were collected from different locations and analyzed compared with farmed *A. officinalis*. Shoots were screened for (i) phenolic compounds by HPLC-DAD and LC-MS; (ii) total phenolic acids and total flavonoid content by the Folin–Ciocalteu and aluminum chloride methods; (iii) vitamin C by HPLC-DAD; (iv) antioxidant activity by the DPPH and ABTS^•+^ methods; and (v) the in vitro antiproliferative activities against HT-29 colorectal cancer cells by the MTT assay. Phenolics ranged from 107.5 (*A. aphyllus*) to 605.4 mg/100 g dry weight (dw) (*A. horridus*). Vitamin C ranged from 15.8 (*A. acutifolius*) to 22.7 mg/100 g fresh weight (fw) (*A. officinalis*). The antioxidant activity was similar in all species, standing out in *A. officinalis* with 5.94 (DPPH) and 4.64 (ABTS) mmol TE/100 g dw. Among phenolics, rutin reached the highest values (574 mg/100 g dw in *A. officinalis*), followed by quercetin, nicotiflorin, asterin, and narcissin. The MTT assay revealed the inhibitory effects of ethanol extracts against HT-29 cancer cells, highlighting the cell growth inhibition exercised by *A. albus* (300 µg/mL after 72 h exposure to cells). This work improves knowledge on the phytochemicals and bioactivities of the shoots of wild *Asparagus* species and confirms their suitability for use as functional foods.

## 1. Introduction

Traditional knowledge about wild edible plants has been seriously threatened for several generations, due to the progressive abandonment of conventional ways of life and the massive exodus of the population from the countryside to the city. Considering that the population is currently aware of the relationship between diet and health and that plant-based diets are increasing among consumers worldwide, it is relevant to boost the potential of wild plants as a source of bioactive compounds to promote their consumption in modern societies, which would contribute to the preservation of traditional knowledge, improve biodiversity, sustainability, and food security, and generate employment opportunities in traditional collecting areas [1,2].

*Asparagus* L. 1753 is a genus belonging to the family Asparagaceae Juss. 1789 (Class Liliopsida Batsch 1802), which includes over 250 species, from which *A. officinalis* L. is the only cultivated species. However, several wild species are traditionally collected for consumption and medicinal purposes in the Mediterranean Basin, such as *A. aphyllus* L., *A. acutifolius* L., and *A. horridus* L. (syn. *A. stipularis* Forssk) [3].

Assessed through color, *A. officinalis* can be eaten as green, white, purple-green, purple-blue, and pink asparagus. White asparagus turns into green asparagus when exposed to sunlight after harvesting. Besides as canned food, these shoots can be marketed as fresh green to be eaten as vegetables [4]. The protein of *Asparagus* contains all essential amino acids in a suitable proportion, and green *Asparagus* contains relatively higher amounts of nutritional components than white ones, except for available carbohydrates. Moreover, this vegetable stimulates intestinal transit due to its high fiber concentration [5].

*Asparagus* shoots, besides being a highly appreciated vegetable due to their appropriate organoleptic and nutritional characteristics, have noticeable functional value since they are reported to contain bioactive compounds, and among these, steroidal saponins are highlighted. Such spears contain vitamins A, B_1_, B_2_, C, and E and folic acid [5], as well as several other healthy bioactive constituents, such as Mg, P, Ca, Fe, flavonoids (kaempferol, quercetin, and rutin), arginine, asparagine, tyrosine, resin, essential oils, and tannin [6].

Among bioactive compounds, phenolics have redox properties, which allow them to act as antioxidants [7]. Flavonoids, including flavones and flavanols, are plant secondary metabolites that have antioxidant activity, which depends on the presence of free OH groups, especially 3-OH. These suppress and scavenge reactive oxygen species, and up-regulate and protect antioxidant defenses. Similarly, phenolics confer oxidative stress tolerance to plant tissues [8].

Although the most widely studied species is the cultivated *A. officinalis*, there is an increasing interest in wild edible *Asparagus* taxa. An exhaustive review on important bioactive compounds of *Asparagus* shoots collected from the wild or cultured in Southern Europe is detailed in Supplementary Table S1. It highlights that *A. acutifolius* has been previously researched in Italy, and it has been reported to be a good source of valuable nutrients including phenolic compounds, carotenoids, and saponins, while its antioxidative and antiproliferative properties were favorable [9,10]. This taxon has been widely scrutinized to establish its biological activities, especially antitumor ones (e.g., [11]). On the other hand, *A. horridus* has been researched looking to establish its antioxidant activity; α-glucosidase inhibitory potential and in vivo protective effect [3]; and anti-inflammatory and anti-cancer activities [12,13]. Among its various phytochemicals, phenolics are highlighted [3]. Most of the research regarding the antioxidant activity of *A. acutifolius* extracts available in the literature was carried out on shoots, which are the edible organs [10,14].

Although the phytochemical composition and bioactivity of cultivated *A. officinalis* and wild *A. acutifolius* are relatively well known, there is a lack of knowledge on the biochemical composition and biological properties of the shoots of *A. horridus*, *A. albus*, and *A. aphyllus*. Moreover, the variability of the phytochemical composition of the various *Asparagus* taxa depending on their ecogeographic locations constitutes an unexplored and interesting task. Therefore, this work was designed to determine in wild and cultivated *Asparagus* species collected in several locations of South Spain valuable phytochemicals, such as vitamin C (Vit C) and phenolic compounds, as well as their in vitro biological activities, such as antioxidant and antitumor ones, seeking to unravel the health benefits of the more relevant shoots from commonly consumed species of this genus.

## 2. Results

### 2.1. Phytochemical Characterization

Moisture, Vit C, total phenolics and flavonoids, and the antioxidant activity measured by the DPPH and ABTS methodologies are detailed in Table 1, the phenolic compound profiles quantified by HPLC-DAD are summarized in Table 2, and the occurrence of phenolic compounds detected in selected *Asparagus* samples by LC-MS is detailed in Supplementary Table S2.

#### 2.1.1. Moisture Content

Moisture ranged from 80.7 in *A. horridus* H1 (from Cabo de Gata) to 91.1 g/100 g in *A. officinalis* O1 (from Láchar). Mean values for the moisture content of *Asparagus* shoots ranged from 84.5 (*A. acutifolius*) to 91.0% (*A. officinalis*).

#### 2.1.2. Vitamin C Content

Table 1 shows the Vit C content of the analyzed *Asparagus* samples. Amounts ranged from 9.5 (AC3, *A. acutifolius* from Navas de San Juan) to 27.1 mg/100 g fw (AC1, *A. acutifolius* from Arroyo Blanco). Mean values for Vit C among *Asparagus* species ranged from 15.8 in *A. acutifolius* to 22.7 mg/100 g fw in *A. officinalis*.

#### 2.1.3. Total Phenolic Compound and Flavonoid Contents

TPC ranged between 238.0 and 468.2 mg GAE/100 g fw in *A. albus* AL2 from Sierra de Cabrera and *A. horridus* H1 from Cabo de Gata. Mean values for *Asparagus* were between 296.6 (*A. albus*) and 402.6 mg GAE/100 g fw (*A. officinalis*). TFC was between 56.9 (*A. aphyllus* AP1, from Alcalá de los Gazules) and 430.8 mg QE/100 g fw (*A. officinalis* O2, from Loja). Mean values for TFC ranged from 96.1 in *A. aphyllus* to 348.3 mg QE/100 g fw in *A. officinalis*.

#### 2.1.4. Antioxidant Activity

The antioxidant activities of the phenolic-containing extracts of *Asparagus* spp. are detailed in Table 1. Values for all samples ranged between 0.72 in *A. albus* AL3 (from Sierra Morena) and 6.08 mmol TE/100 g dw in *A. officinalis* O2 (from Loja) according to the DPPH assay. Considering species, the antioxidant activity was between 2.34 (*A. albus*) and 5.94 mmol TE/100 g dw (*A. officinalis*). In the case of ABTS^•+^, there were no significant differences in the mean values among the different *Asparagus* taxa. Considering samples, the lowest and highest antioxidant activities were recorded for *A. horridus* H3 (1.86 mmol TE/100 g dw) and *A. officinalis* O1 (4.23 mmol TE/100 g dw). Overall, the highest antioxidant activities determined by the DPPH and ABTS^•+^ methods were recorded in cultivated *A. officinalis*. The antioxidant activity of pure compounds assessed with comparative purposes was between 17.54 and 23.34 mmol of TE/100 g for α-tocopherol and ascorbic acid (DPPH methodology), while for the ABTS^•+^ assay, the values ranged between 8.78 (α-tocopherol) and 10.43 mmol TE/100 g (ascorbic acid).

#### 2.1.5. Phenolic Compound Profiles

The phenolic compound profiles and total phenolic contents quantified by HPLC-DAD are detailed in Table 2, and those detected for selected *Asparagus* species by LC-MS are listed in Supplementary Table S2. Chromatograms were screened at 254, 280, and 320 nm (Supplementary Table S3), where they showed good molar extinction coefficients. The phenolic compounds were identified by comparing the recorded absorption spectra of all peaks from the various chromatograms with pure standards. Quantified phenolic compounds were rutin, asterin, nicotiflorin, narcissin, and quercetin, and values for these were characteristic of each species. Rutin ranged from 59.5 (*A. aphyllus*) to 574.3 mg/100 g dw (*A. officinalis*); nicotiflorin was between 8.4 (*A. albus*) and 72.5 mg/100 g dw (*A. horridus*); asterin ranged from 1.3 (*A. albus*) to 33.2 mg/100 g dw (*A. acutifolius*); and narcissin and quercetin were within ~5–15 mg/100 g dw for all studied species, but narcissin was below the detection limit in *A. officinalis*.

The structures of all identified compounds that occurred in *Asparagus* shoots were confirmed by LC-MS. The precision/injection repeatability test showed good precision in peak area (standard deviation < 1%) and peak retention time (±2%). The results of the linearity range, the regression equation, LOD, LOQ, and recoveries for all quantified compounds are presented in Supplementary Table S3. Besides the compounds quantified with the HPLC-DAD system, several phenolic compounds were identified using the LC-MS system from the *m*/*z* value of the molecular ion, but some of these compounds could not be clearly attributed to any of the chromatographic peaks obtained with the HPLC-DAD system. 

### 2.2. Antitumor Activity

**Figure 1:**
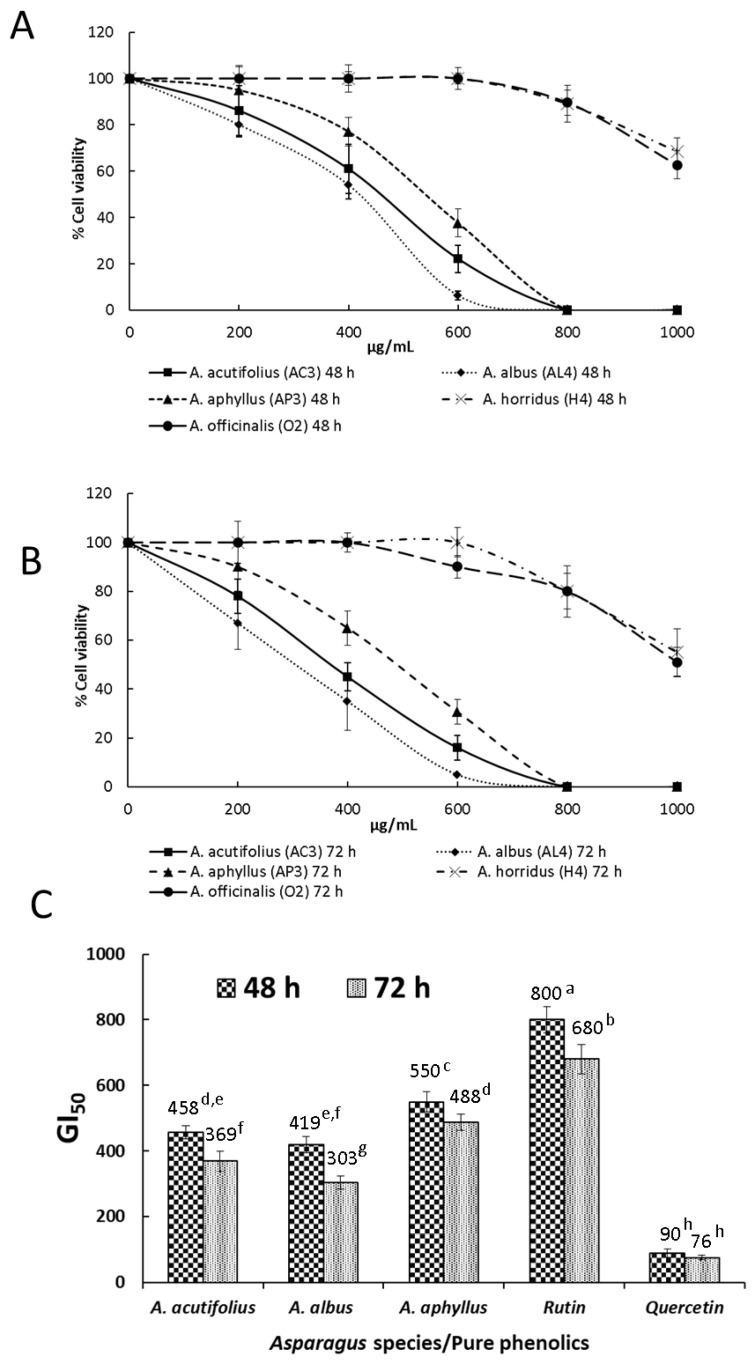
MTT assay. (**A**) Dose–response curves of HT-29 cell viability after treatment with different concentrations of ethanol extracts of *Asparagus* shoots for 48 h. (**B**) Dose–response curves of HT-29 cell viability after treatment with different concentrations of ethanol extracts of *Asparagus* shoots for 72 h. (**C**) GI_50_ of HT-29 cells after treatment with ethanol extracts of *Asparagus* shoots, rutin, and quercetin for 48 and 72 h. The GI_50_ value is detailed over each column. Data represent the mean of three complete independent experiments ± SD (error bars). In a bar, means followed by different letters are significantly different at *p* < 0.05.

The MTT assay was accomplished to evaluate the inhibitory effects of selected *Asparagus* extracts on HT-29 human colorectal cancer cell viability. Extracts having the highest antioxidant activity (one of each species) were selected for this assay. Figure 1A,B show the activity of such extracts against cancer cell viability after 48 and 72 h of treatment, respectively. Cell growth inhibition was exercised much better by *A. acutifolius*, *A. albus*, and *A. aphyllus,* which at 600 µg/mL and 72 h of cell exposure induced 22.1, 6.2, and 37.6% of cancer cell viability in comparison with controls without extract addition. GI_50_ values, i.e., the doses of extracts that inhibited cell growth by 50%, of selected samples and those of two pure phenolic compounds occurring in samples are shown in Figure 1C. After a 72 h incubation period, GI_50_ for *A. acutifolius* (AC4), *A. albus* (AL4), *A. aphyllus* (AP3), rutin, and quercetin were 369, 303, 488, 680, and 76 µg/mL, respectively.

## 3. Discussion

### 3.1. Vitamin C Content

Vit C, i.e., ascorbic acid, is a water-soluble vitamin found in certain foods. It has a role as an antioxidant in the human organism, helping to protect cells against damage caused by free radicals and allowing the synthesis of collagen [15,16]. The mean values of Vit C were quite similar for the different analyzed species, and the highest value was obtained in cultivated *A. officinalis* (22.7 mg/100 g fw). The remaining species showed Vit C content in the ~16–17 mg/100 g fw range. It is not surprising that the Vit C content in cultivated *Asparagus* was higher than that in wild ones, considering that cultivated plants receive a greater supply of nutrients and are not subjected to environmental stress. On the other hand, it has been suggested that Vit C in cultivated *Asparagus* depends mainly on the harvesting season [17]; however, in the case of wild *Asparagus,* it is difficult to determine it, since the various samples were collected from locations having different ecogeographical features.

Some studies suggest that the features of the cultivation area influence the chemical composition of *Asparagus* shoots. Hence, the Vit C content of eight white *A. officinalis* varieties cultivated in Greece was 14.1–20.2 mg/100 g fw [18], in line with the results of this work. Moreover, an investigation on the chemical composition of wild *A. acutifolius* shoots from Portugal and its comparison with similar samples from different origins concluded that there were great differences in Vit C contents [19].

### 3.2. Phenolic Compound Content

TPC values showed no significant differences among species when assessed through the F-C method (Table 1), although a wide range of variation within species depending on the collecting area was noted. For instance, TPC ranged between 238.0 and 515.2 mg GAE/100 g fw in *A. albus.* TFC was typically half of TPC in most cases, and a wide variation in TFC was also noted within species; for instance, *A. officinalis* showed significantly higher values than those of *A. aphyllus*.

In this study, most *Asparagus* shoots, all of them collected in Southern Spain, showed significant amounts of phenolic compounds. This fact can be due to the stressful conditions of a warm-summer Mediterranean climate, which can activate a multi-gene response that leads to changes in secondary metabolite accumulation, including the synthesis of phenolic compounds [20,21]. Phenolics are chemical mediators between plants and their environment, and consistently, both phenolics and antioxidant compounds in *Asparagus* vary widely depending on the phenological stage, environmental conditions (seasonal changes, radiation, soil type, and water availability), and chemotypes [22,23].

TPC and TFC of the shoots of three *Asparagus* species analyzed in the current work have been previously reported (Supplementary Table S1). Data from *A. acutifolius* and *A. officinalis* showed a wider range of variation than that obtained here. Such variation can be due to climatic features, analytical procedures, different varieties under study, and the analysis of shoots having different stages of development. The mean values obtained here had intermediate values when compared with those of Supplementary Table S1 for *A. acutifolius* (TPC 352.9 mg GAE/100 g fw and TFC 257.6 mg QE/100 g fw) and for *A. officinalis* (TPC 402.6 mg GAE/100 g fw and TFC 348.3 mg QE/100 g fw). Regarding *A. horridus*, Adouni et al. [3] reported slightly lower values for TPC and TFC than those found in this work (398.4 mg GAE and 143.6 mg QE by 100 g fw).

The phenolic profiles of all samples analyzed and quantified by HPLC-DAD are given in Table 2, while the compounds identified by LC-MS are detailed in Supplementary Table S2. Notice that there were several phenolic compounds identified by their *m*/*z* ions (Supplementary Table S3) but lacking quantification considering an absence of specific peaks attributed in the HPLC-DAD chromatograms. Overall, the TPC obtained by the sum of quantified phenolics by HPLC-DAD was significantly lower than that obtained by the F-C methodology (which provides GAE). This fact is explained by the following: (i) the F-C method informs on total phenolic compounds, while chromatographic methods report only the concentration of identified compounds; and (ii) there is an overestimation of the actual phenolic content of the F-C method due to its unspecificity toward targeted phenolics [24].

The identification of peaks detected with the LC-MS system was based on the *m*/*z* ion. For instance, the compound with *m*/*z* = 595 and MS^2^ ion at 271 due to a loss of 324 amu corresponding to two glucose moieties was confirmed as pelargonidin 3-*O*-diglucoside. As the ion at 271 is the most abundant one in MS^2^, it indicates that the two glucose moieties are linked to each other and correspond to a diglucoside derivative of pelargonidin. Among others, it highlights the occurrence of chelidonic acid, which is a heterocyclic organic acid with a pyran skeleton, which was detected in all samples; chlorogenic acid (an ester of caffeic acid and (−)-quinic acid) was absent in *A. horridus*; *p*-coumaric acid hexoside (a hydroxycinnamic acid) and the flavonoids nicotiflorin, rutin, and quercetin were present in all samples; syringic acid (a gallic acid derivative); and 5-*O*-*p*-coumaroyl quinic acid (a cinnamate ester), (−)-epicatechin gallate (a flavan-3-ol, a type of flavonoid), and isoquercetin (a flavonoid) were restricted to *A. albus* samples. The remaining phenolics had a more random distribution among the different species. In all taxa, rutin reached the highest values, especially in *A. officinalis* and *A. acutifolius*, followed by nicotiflorin, which had the highest values in *A. horridus*, while narcissin was undetected in *A. officinalis*. Asterin and quercetin were minor contributors to this profile. This situation agrees with previously reported values (Supplementary Table S1), although it can be noted that a great variety of phenolics was previously reported in minor amounts. The concentration obtained for rutin in *A. officinalis* agrees with most values given by various authors, such as Motoki et al. [25], and such an amount suggests that wild edible shoots can be used as functional foods, considering the bioactivity of this flavonoid. It develops potent antioxidant and anti-inflammatory actions and prevents neurodegenerative and cardiovascular disorders, as well as skin cancer, among other diseases [26].

It was reported that medicinal plants having high amounts of phenolic compounds develop potent antioxidant actions [27], and ethanol is a suitable solvent for their extraction given that polyphenols are linked to the cell-wall matrix through a glycosidic/ester linkage; thus, alcohol-based solvents are appropriate for the extraction of all types of phenolic compounds [28]. Then, this solvent was selected for extraction in this study, and it might be used in the food industry for rutin-rich extract obtainment.

### 3.3. Antioxidant Activity

Two different methodologies were accomplished in this work for assessing this property, and differences in values obtained from both methods were noted. DPPH· is a relatively stable nitrogen radical, whereas ABTS^•+^ is more unstable and is produced upon reaction with potassium persulfate. It has been reported that some compounds that react rapidly with peroxyl radicals do not react well with the DPPH radical, due to steric hindrance between the compounds [29]. The results obtained by the two methods are shown in Table 1. Overall, the results of this study agree with the highest values reported for both *A. acutifolius* and *A. officinalis* (Supplementary Table S1, e.g., [30,31]). The lowest antioxidant activity determined by the DPPH· and ABTS^•+^ methods was recorded in some samples of *A. albus*, and the highest one was obtained in *A. officinalis* samples. The results obtained by the ABTS^•+^ and DPPH methods for *A. officinalis* samples were higher than those reported by Sun et al. [31] for juice samples of the same species (DPPH 1.74 and ABTS^•+^ 2.64 mmol TE/100 g) and those reported by Fan et al. [29] for *A. officinalis* residues (ABTS^•+^ 1.42 and DPPH 0.62 mmol TE/100 g). Such differences in bioactivity could be because plants grown under different environmental conditions were analyzed.

The relationship between phenolic compound content and antioxidant activity has been extensively studied [32,33], and the antioxidant activity found in *Asparagus* shoots has been attributed to flavonoids [34]. Kulczyński et al. [35] identified a positive correlation between ABTS^•+^ and phenolic compounds in *A. officinalis* samples; however, this relationship changes according to the type of phenolic compound considered, and quercetin and rutin showed the highest values. Fan et al. [29] evidenced a positive correlation between antioxidant activity determined by the ABTS^•+^ and DPPH methods and both TPC and TFC, being higher for ABTS^•+^, due to the above-exposed reasons. Moreover, the ABTS^•+^ radical is more soluble in organic compounds and water than DPPH; thus, the ABTS^•+^ method may have higher sensitivity in samples with high water content, such as *Asparagus* samples [36].

Recently, it has been demonstrated that different cultivation systems have a significant effect on the bioactive compounds, antioxidant enzymes, and anti-cancer activity of *A. officinalis* [37]. In this research, spears grown in an open field (OF) and a rain-shelter house (RSH) system were analyzed. Results showed that rutin and *trans*-*p*-coumaric acid contents, as well as the ABTS^•+^ and DPPH radical scavenging activity of *Asparagus* spears grown in the OF, were higher than those grown in the RSH system. Given that the samples analyzed in the present work were collected from the wild, i.e., OF, this factor would presumably not affect the results obtained here.

The antioxidant activity of pure compounds (α-tocopherol, ascorbic acid, and caffeic acid) was also checked with comparative purposes, and the results obtained by the DPPH method were approximately double those of the ABTS^•+^ procedure, although values obtained by each method for all these compounds were quite similar (Table 1). Interestingly, the highest antioxidant activity, which was found in *A. officinalis*, was approximately between half and one-third of that showed by such pure molecules by the DPPH method and approximately half when checked by the ABTS^•+^ methodology. Other *Asparagus* species showed much lower values, but *A. aphyllus* had also a high antioxidant activity, approximately half that of *A. officinalis*.

**Figure 2:**
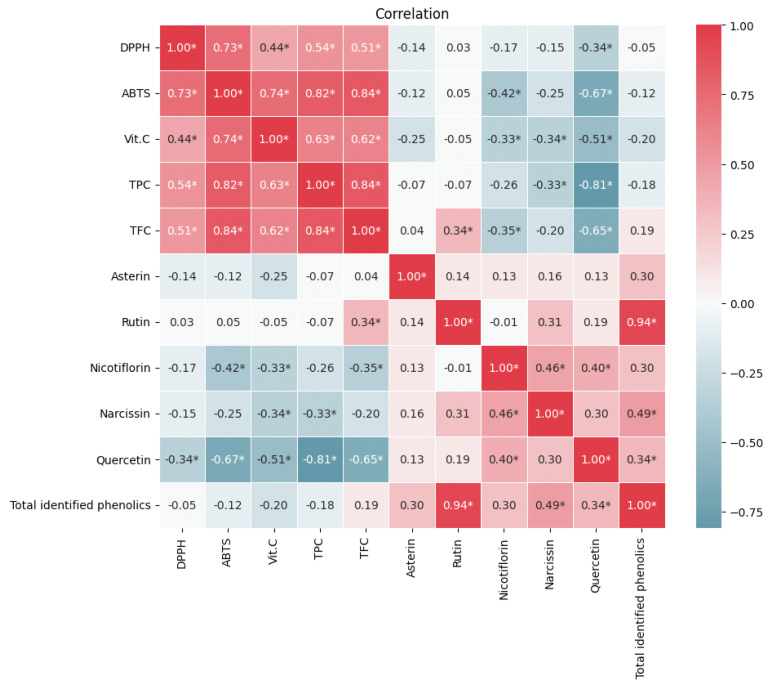
Heat map of the correlation between the different variables. The redder colors indicate a stronger and positive correlation, while the bluer colors indicate a stronger and negative correlation. Correlations were evaluated with a significance of *p* < 0.05 (*).

Figure 2 shows the correlations between the antioxidant activity and bioactive compounds (TPC, TFC, Vit C, rutin, quercetin, rosmarinic acid, and total identified phenolics) in *Asparagus* samples. Notice that TFC, TPC, and Vit C have a high and positive correlation with the antioxidant activity determined by the ABTS^•+^ method. This correlation indicated that the compounds that have significant reducing ability quantified by the F-C methodology and Vit C contributed largely to the antioxidant activity of the various *Asparagus* samples.

### 3.4. Antiproliferative Activity of the Ethanol Extracts of Asparagus Shoots on HT-29 Cancer Cells

The antitumor actions of *Asparagus* extracts on cancer cells constitute a hot topic. Previous studies indicated that saponins from *Asparagus* are responsible for a reduced risk of colorectal cancer by inducing cytotoxicity and apoptosis [38,39]. Recently, the synergistic action of an *A. officinalis* extract combined with paclitaxel at low concentrations has been evidenced, which inhibited cell proliferation and induced apoptosis in paclitaxel-sensitive and -resistant ovarian cancer cell lines. Such treatment exercised DNA damage and suppressed microtubule dynamics [40].

The antitumor activities of different *A. acutifolius* organs were previously tested in human cancer cell lines (HCT-116, colon) and (HepG2, liver) using the MTT assay [11], and antitumor activities were characteristic for tested organs. The ethanol extract of the rhizome showed activity against both cell lines tested, while the leaf extract was more cytotoxic than the rhizome. However, the stem and pericarp extracts lack antitumor activity. Likewise, the rhizome from *A. acutifolius* showed an important cytotoxic activity against the HepG2 cell line, and similar values were reported by other authors for *A. adscendent* [41] and *A. filicinius* [42], who related this activity to the presence of saponins and their genins. As for *A. albus*, low IC_50_ values (40 µg/mL) were found after HCT-116 colon cancer cell exposure to the rhizome extracts, while the leaf extracts had similar IC_50_ values against such cells [43].

Adouni et al. [3] reported for the hydroalcoholic extract of *A. horridus* shoots in cell cultures GI_50_ of 298.63 in MCF-7 (breast carcinoma); 244.26 in NCI-H460 (non-small-cell lung cancer); 208.24 in HeLa (cervical carcinoma); 200.77 in HepG2 (hepatocellular carcinoma); and >400 µg/mL in PLP2 (liver primary culture).

Among the various antitumor phytochemicals contained in different organs of *Asparagus,* polysaccharides are highlighted. The antitumor activity of *Asparagus* tissues was partially related to the presence of polysaccharides, which also exhibit antioxidant activity. Then, it is likely that the polysaccharides present in the extracts tested in this study against HT-29 cells contributed, at least partially, to the noted activity. These molecules demonstrated significant inhibition of human cervical (HeLa) and liver (BEL-7404) cancer cell lines after protein elimination [44]. Moreover, among the various pectic polysaccharide sub-fractions from *Asparagus*, the fractions with the higher degree of esterification showed a stronger immunomodulatory activity on RAW 264.7 macrophages [45].

Interestingly, it has been reported that the cultivation system of *A. officinalis* had a significant effect on the survival rate of MCF-7 breast cancer cells treated with *Asparagus* shoot extracts. Cells treated with extracts of plants grown in an OF had a lower survival rate than cells treated with extracts from plants grown in the RSH system. However, cultivation systems lack effects on quercetin, sinapic acid, ferulic acid, and caffeic acid contents, as well as catalase activity and Calu-6 cell viability [37].

Given the nature of the extracts tested in this work against HT-29 cells (ethanol 96%), it is likely that besides phenolic compounds, some saponins and polysaccharides were present in such extracts. After 48 and 72 h of treatment (Figure 1A and Figure 1B, respectively), the MTT assay revealed concentration- and time-dependent inhibitory effects on HT-29 cells for all assayed extracts. Cell viability after 72 h of treatment at the maximum concentration tested (1000 μg/mL) and for the different species was 15–20% higher than that obtained at 48 h.

After 72 h cell culture, cell growth inhibition was exercised much better by *A. acutifolius* (AC3), *A. albus* (AL4), and *A*. *aphyllus* (AP3), which reached GI_50_ of between 300 and 500 µg/mL of extract, with null cell viability at 800 µg/mL, and GI_50_ were 369, 303, and 488 µg/mL, respectively. The antiproliferative activity against HT-29 cells of some pure phenolic compounds occurring at high concentrations in *Asparagus* extracts was also checked. Rutin and quercetin showed GI_50_ values of 680 and 76 µg/mL. Considering that rutin was a major phenolic present in the ethanol extract and its low activity, the recorded antitumor activity was likely due to a synergy between polysaccharides, saponins, and some phenolic compounds, i.e., quercetin, as previously described in *Buglossoides* seed extracts when tested against HT-29 cancer cells, since these cells are not very sensitive to phenolics [46]. In contrast to rutin, quercetin has been previously demonstrated to inhibit colon cancer cell growth through the induction of apoptosis. It decreases the expression of Bcl-2, a protein that acts as an inhibitor of programmed cell death [47]. Even though the concentration of rutin is much higher than that of quercetin in all checked species, given the low GI_50_ of the latter (76 µg/mL), it could have influenced the noted antitumor activity. However, quercetin concentrations vary slightly between the different samples, so the antitumor effects would probably be due to a synergy among several compounds present in the ethanol extract, as previously mentioned.

## 4. Materials and Methods

### 4.1. Reagents and Chemicals

Unless otherwise indicated, all chemicals and solvents were purchased from Merck (Madrid, Spain). L-Ascorbic acid was obtained from Labkem (Barcelona, Spain). Aluminum chloride and sodium carbonate were obtained from Sigma-Aldrich Co. (St Louis, MO, USA). Sodium nitrite, sodium hydroxide, and oxalic acid were purchased from Panreac (Barcelona, Spain). Water was purified using a Milli-Q system (Millipore, Burlington, MA, USA). All the chemicals used, including the solvents, were of analytical grade.

### 4.2. Samples

Data on shoots collected for this work are shown in Table 3. Upon arrival at the laboratory, the shoots were labeled, weighed, measured, and frozen at −20 °C until analysis. Approximately 2 g of each fresh sample was used for moisture analysis, which was carried out in a forced air oven at 100 °C until constant weight. Just before analysis, shoots were ground with a mortar. 

### 4.3. Extraction of Phenolic Compounds from Asparagus Species

This methodology is fully detailed in Supplementary File S1. Extraction and analysis of phenolic compounds from *Asparagus* species were carried out according to Lyashenko et al. [48].

### 4.4. Characterization of Phenolic Compounds by HPLC-DAD

**Figure 3:**
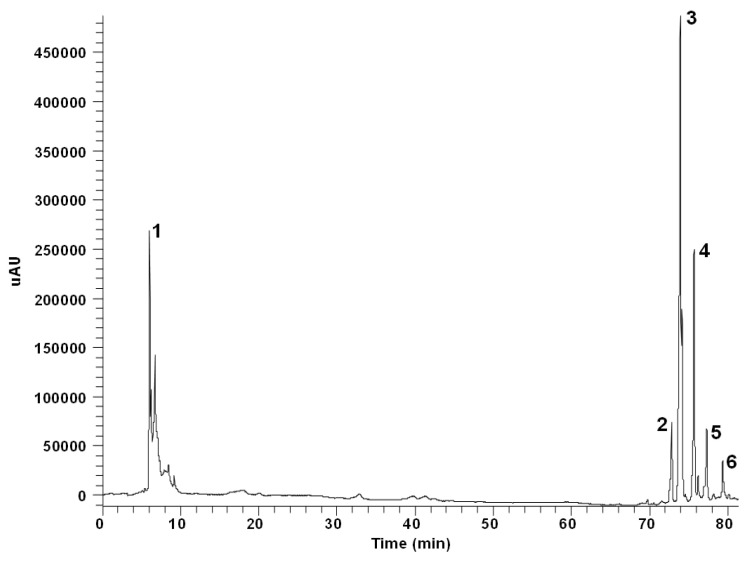
A 254 nm-HPLC chromatogram of the phenolic-containing ethanol extract of *Asparagus horridus* (H2). 1. Ascorbic acid. 2. Asterin. 3. Rutin. 4. Nicotiflorin. 5. Narcissin. 6. Quercetin.

This methodology is fully detailed in Supplementary File S1. HPLC analyses of phenolics were carried out using a Finnigan Surveyor chromatograph equipped with a diode-array detector (DAD) and a reverse-phase C18 column (Hypersil Gold, 250 × 4.6 mm i.d., 5 µm particle size) (Thermo Electron, Cambridge, UK). The compounds were separated with a gradient elution using acidified water (1% acetic acid) (A) and acetonitrile (B) as mobile phase at 25 °C. The running time was 100 min. A 254 nm-HPLC-DAD chromatogram of *A. horridus* (H2) is depicted in Figure 3. Quantification of the compounds was made using external calibration curves obtained from pure standards (Sigma-Aldrich, St. Louis, MO, USA) in the HPLC-DAD system.

### 4.5. Characterization of Phenolic Compounds by LC-MS

This methodology is fully detailed in Supplementary File S1, and the HPLC-DAD and LC-MS parameters for the analysis of phenolic-rich extracts of *Asparagus* shoots are detailed in Supplementary Table S3. The chromatographic separations were performed on a Vanquish Flex Quaternary LC equipped with a reverse-phase C18 column (Hypersil Gold, 100 mm × 2.1 mm, 1.9 μm) at a flow rate of 0.2 mL/min. The total running time was 39 min. The LC system was coupled to a hybrid mass spectrometer Q-Orbitrap Thermo Fisher Scientific using electrospray ionization (ESI) in positive and negative ion mode. 

### 4.6. Determination of Total Phenolic Content

Total phenolic content (TPC) was measured using the F-C assay, as reported by Singleton et al. [49] with minor modifications. This methodology is fully detailed in Supplementary File S1. The results were expressed as mg of gallic acid equivalents (GAE) per 100 g of fw using a standard curve of GA. Determinations were performed in triplicate.

### 4.7. Determination of Total Flavonoid Content

Total flavonoid content (TFC) of *Asparagus* samples was determined according to Zou et al. [50] with some modifications, and this methodology is fully detailed in Supplementary File S1. The results were expressed as mg of quercetin equivalents (QE) per 100 g fw using a standard curve of quercetin (10–500 μg/mL). Determinations were performed in triplicate.

### 4.8. Extraction and Quantification of Vitamin C

Vit C (L-ascorbic acid) content was determined according to Volden et al. [51] with minor modifications. This methodology is fully explained in Supplementary File S1. The analysis of Vit C was carried out using the previously described HPLC-DAD system. Ascorbic acid was quantified by external calibration, and results were recorded as mg/100 g fw. All data are presented as mean ± standard deviation of samples analyzed in triplicate.

### 4.9. Antioxidant Activity

Extraction was carried out with ethanol (96%) according to the methodology described by Forbes-Hernández et al. [52] with some modifications. This methodology is fully explained in Supplementary File S1. The antioxidant activity using the ABTS method was determined using a solution of ABTS^•+^ radical 2,2′-azinobis (3-Ethylbenzothiazoline-6-sulfonic acid) in ethanol (2.45 mM). The DPPH method was carried out according to Skenderidis et al. [53], and is fully described in Supplementary File S1. The absorbance of the solution was measured at 517 nm. The values of ABTS^•+^ and DPPH were expressed as mmol of Trolox Equivalent/100 g dw (mmol TE/100 g dw).

### 4.10. Antitumor Assay

This methodology is fully detailed in Supplementary File S1. The antiproliferative activity of the 96%-ethanol extract from *Asparagus* shoots was assayed on the HT-29 human colon cancer cell line as described by Lyashenko et al. [48]. 

### 4.11. Statistical Analysis

All samples were analyzed in triplicate. Data were assessed for normality using a Shapiro–Wilk test and submitted to one-way ANOVA, and the comparison of means was made using Duncan’s multiple-range test. Statistical analyses were performed using Statgraphics^©^ Centurion XVI (StatPoint Technologies, Warrenton-Virginia, VA, USA).

## 5. Conclusions

The levels of phytochemicals and biological activities found in the shoots of four wild *Asparagus* species lead us to consider them as functional foods. Cultured *A. officinalis* is highlighted considering its values for Vit C, TPC, TFC, antioxidant activity, rutin, and total phenolics quantified through the HPLC-DAD system. This fact was probably due to that farmed plants receive a controlled supply of water and nutrients, which prevents stressful situations. Therefore, it would be expected that agronomic research on the four wild taxa analyzed here could lead to equalizing the phytochemical content and the biological activities of cultured *A. officinalis*. TFC, TPC, and Vit C have a clear and positive correlation with the antioxidant activity determined by the ABTS^•+^ method, which indicated that the compounds quantified by the F-C methodology and Vit C contributed largely to the high antioxidant activity detected in the various *Asparagus* samples. Regarding phenolics, rutin reached the highest values in all analyzed species, especially in *A. officinalis* and *A. acutifolius*, followed by nicotiflorin, which had the highest values in *A. horridus*, while narcissin was undetected in *A. officinalis*. The LC-MS system allowed the detection of several phenolic glycosides, and some of them had a characteristic distribution among species. Extracts from wild *Asparagus* species (*A. acutifolius*, *A. albus*, and *A. aphyllus*) showed higher antitumor activity against HT-29 cells than that of cultured *A. officinalis*. Future research should be focused on the study of other phytochemicals, such as sterols and tocols, and checking the phytochemicals occurring in wild *Asparagus* species after they have been cultivated in optimized farming systems. Furthermore, extensive research on the bioactivity of the various *Asparagus* extracts against other cell lines, including normal colorectal cells, and the mechanisms through which the extracts exercise antitumor actions will be welcomed.

## Figures and Tables

**Table 1:** Moisture, antioxidant activity, vitamin C, and total phenolic and flavonoid content of *Asparagus* samples ^1,2,3^.

Samples/Codes	Moisture g/100 g	Antioxidant Activity		Vitamin C mg/100 g fw	TPC mg GAE/100 g fw	TFC mg QE/100 g fw
		DPPH mmol TE/100 g dw	ABTS mmol TE/100 g dw			
*A. acutifolius*						
AC1	81.4 ± 0.0 ^j^	3.13 ± 0.01 ^i^	1.97 ± 0.04 ^k^	27.1 ± 4.1 ^a^	320.2 ± 4.6 ^f^	189.2 ± 19.5 ^efg^
AC2	84.6 ± 0.3 ^gh^	2.07 ± 0.05 ^l^	2.58 ± 0.18 ^fgh^	13.4 ± 2.9 ^cde^	398.5 ± 24.8 ^cd^	306.9 ± 24.8 ^bc^
AC3	85.2 ± 0.3 ^fg^	4.20 ± 0.01 ^e^	2.87 ± 0.11 ^de^	9.5 ± 1.5 ^e^	398.3 ± 8.8 ^cd^	334.2 ± 25.0 ^b^
AC4	86.9 ± 0.3 ^de^	2.55 ± 0.05 ^j^	2.80 ± 0.03 ^def^	13.3 ± 1.0 ^cde^	294.4 ± 19.1 ^fg^	200.1 ± 27.7 ^e^
Mean ± SD	84.5 ± 2.3 ^B^	2.99 ± 0.92 ^B^	2.56 ± 0.41 ^B^	15.8 ± 7.7 ^A^	352.9 ± 53.6 ^A^	257.6 ± 73.7 ^AB^
*A. albus*						
AL1	88.0 ± 0.2 ^bcd^	3.49 ± 0.02 ^g^	2.80 ± 0.06 ^def^	18.7 ± 6.7 ^abcd^	289.2 ± 4.3 ^g^	159.2 ± 8.6 ^fgh^
AL2	88.1 ± 0.2 ^bcd^	0.98 ± 0.03 ^m^	2.02 ± 0.11 ^jk^	13.5 ± 1.0 ^cde^	238.0 ± 1.7 ^h^	58.2 ± 6.8 ^k^
AL3	86.4 ± 1.2 ^ef^	0.72 ± 0.02 ^n^	2.28 ± 0.06 ^ij^	22.2 ± 7.1 ^abc^	413.2 ± 4.0 ^cd^	276.3 ± 7.5 ^cd^
AL4	89.0 ± 0.5 ^b^	4.17 ± 0.03 ^e^	2.94 ± 0.05 ^de^	9.9 ± 2.1 ^de^	245.9 ± 6.5 ^h^	168.2 ± 5.1 ^efgh^
AL5	88.8 ± 0.4 ^bc^	3.90 ± 0.16 ^f^	3.78 ± 0.06 ^c^	18.0 ± 2.5 ^abcde^	515.2 ± 21.7 ^a^	304.9 ± 34.5 ^bcd^
Mean ± SD	88.1 ± 1.0 ^AB^	2.7 ± 1.7 ^B^	2.8 ± 0.7 ^B^	16.5 ± 4.8 ^A^	340.3 ± 120.3 ^A^	193.4 ± 99.2 ^AB^
*A. aphyllus*						
AP1	83.7 ± 0.9 ^hi^	3.05 ± 0.01 ^i^	2.30 ± 0.03 ^hi^	13.8 ± 3.6 ^cde^	300.6 ± 2.1 ^fg^	56.9 ± 5.5 ^k^
AP2	85.3 ± 0.5 ^fg^	4.57 ± 0.02 ^d^	2.79 ± 0.05 ^def^	13.5 ± 2.2 ^cde^	360.6 ± 15.7 ^e^	92.4 ± 3.5 ^jk^
AP3	86.5 ± 1.2 ^ef^	4.67 ± 0.01 ^c^	3.02 ± 0.11 ^d^	24.2 ± 5.3 ^ab^	355.3 ± 9.5 ^e^	139.1 ± 3.8 ^hi^
Mean ± SD	85.2 ± 1.4 ^B^	4.10 ± 0.91 ^AB^	2.70 ± 0.37 ^B^	17.2 ± 6.1 ^A^	338.8 ± 33.2 ^A^	96.1 ± 41.2 ^B^
*A. horridus*						
H1	80.7 ± 1.4 ^j^	3.48 ± 0.01 ^g^	1.92 ± 0.01 ^k^	11.6 ± 5.3 ^de^	468.2 ± 0.5 ^b^	151.0 ± 14.6 ^gh^
H2	84.0 ± 0.7 ^ghi^	2.33 ± 0.01 ^k^	2.42 ± 0.07 ^ghi^	21.1 ± 2.8 ^abc^	362.3 ± 10.7 ^e^	128.7 ± 2.3 ^hij^
H3	83.3 ± 1.1 ^i^	3.03 ± 0.01 ^i^	1.86 ± 0.02 ^k^	15.3 ± 0.6 ^bcde^	418.5 ± 19.8 ^cd^	135.5 ± 2.4 ^hi^
H4	87.5 ± 0.1 ^cde^	3.49 ± 0.01 ^g^	2.67 ± 0.10 ^efg^	11.1 ± 4.8 ^de^	423.1 ± 12.0 ^c^	195.4 ± 19.5 ^ef^
H5	87.3 ± 0.5 ^de^	3.30 ± 0.01 ^h^	3.05 ± 0.02 ^d^	22.0 ± 7.1 ^abc^	319.7 ± 20.7 ^f^	107.4 ± 18.0 ^ij^
Mean ± SD	84.6 ± 2.9 ^B^	3.13 ± 0.48 ^B^	2.38 ± 0.50 ^B^	16.2 ± 5.5 ^A^	398.4 ± 57.8 ^A^	143.6 ± 32.9 ^B^
*A. officinalis*						
O1	91.1 ± 0.2 ^a^	5.81 ± 0.03 ^b^	4.23 ± 0.49 ^b^	21.8 ± 7.3 ^abc^	393.0 ± 17.7 ^d^	265.7 ± 28.0 ^d^
O2	90.9 ± 0.3 ^a^	6.07 ± 0.09 ^a^	5.05 ± 0.03 ^a^	23.5 ± 4.5 ^ab^	412.1 ± 9.4 ^cd^	430.8 ± 38.6 ^a^
Mean ± SD	91.0 ± 0.1 ^A^	5.94 ± 0.18 ^A^	4.64 ± 0.58 ^A^	22.7 ± 1.2 ^A^	402.6 ± 13.5 ^A^	348.3 ± 116.7 ^A^
α-Tocopherol	-	17.54 *±* 0.57	8.78 ± 0.45	-	-	-
Ascorbic acid	-	23.34 *±* 1.43	10.43 ± 0.16	-	-	-
Caffeic acid	-	22.18 *±* 0.32	10.34 ± 0.29	-	-	-

^1^ Data represent means ± SD of samples analyzed in triplicate. ^2^ Differences in moisture, antioxidant activity, vitamin C, and phenolic compounds of the various samples were tested according to one-way ANOVA followed by Duncan’s multiple-range test. ^3^ Within a column, means followed by different lowercase letters are significantly different at *p* < 0.05, and means followed by capital letters represent the ANOVA test effected for mean values of species (*p* < 0.05).

**Table 2:** Phenolic compounds of the ethanol extracts of *Asparagus* shoots ^1,2,3^.

Samples/ Codes	Phenolic Compounds (mg/100 g dw)					
	Asterin ^4^	Rutin	Nicotiflorin ^5^	Narcissin ^5^	Quercetin	Total Identified Phenolics
*A. acutifolius*						
AC1	n.d.	303.6 ± 30.5 ^e^	6.6 ± 0.2 ^ij^	4.7 ± 0.5 ^fg^	14.4 ± 0.6 ^cd^	329.3 ± 30.5 ^de^
AC2	81.6 ± 7.7 ^a^	310.5 ± 62.0 ^de^	12.4 ± 1.2 ^hi^	11.6 ± 0.5 ^cd^	11.3 ± 0.6 ^efg^	427.4 ± 62.5 ^c^
AC3	8.1 ± 1.6 ^ef^	618.9 ± 64.3 ^ab^	35.7 ± 3.6 ^de^	31.4 ± 4.7 ^b^	14.3 ± 1.0 ^cde^	708.4 ± 64.6 ^a^
AC4	10.0 ± 2.9 ^e^	292.9 ± 64.4 ^ef^	37.3 ± 5.7 ^d^	0.7 ± 0.1 ^h^	17.8 ± 0.9 ^ab^	358.7 ± 64.7 ^cd^
Mean	33.2 ± 41.9 ^A^	381.5 ± 158.4 ^AB^	23.0 ± 15.8 ^A^	12.1 ± 13.6 ^A^	14.5 ± 2.7 ^A^	456.0 ± 173.2 ^A^
*A. albus*						
AL1	0.5 ± 0.2 ^g^	243.8 ± 61.2 ^efg^	11.8 ± 0.2 ^i^	2.7 ± 0.7 ^gh^	14.9 ± 0.7 ^bc^	273.7 ± 61.2 ^def^
AL2	2.0 ± 0.3 ^g^	397.6 ± 64.5 ^d^	7.9 ± 1.0 ^ij^	5.9 ± 0.1 ^fg^	14.4 ± 0.3 ^cd^	427.8 ± 64.5 ^c^
AL3	n.d.	278.7 ± 52.7 ^ef^	7.3 ± 0.9 ^ij^	3.1 ± 0.3 ^gh^	10.6 ± 1.0 ^fg^	299.7 ± 52.7 ^def^
AL4	n.d.	536.7 ± 55.4 ^bc^	12.9 ± 0.5 ^hi^	9.6 ± 1.5 ^cde^	14.5 ± 0.7 ^cd^	573.7 ± 55.4 ^b^
AL5	n.d.	28.4 ± 8.8 ^j^	2.3 ± 0.4 ^j^	n.d.^f^	3.9 ± 0.8 ^h^	34.6 ± 8.8 ^g^
Mean	1.3 ± 1.1 ^A^	297.0 ± 189.0 ^ABC^	8.4 ± 4.2 ^A^	5.3 ± 3.2 ^A^	11.7 ± 4.7 ^A^	321.9 ± 200.0 ^AB^
*A. aphyllus*						
AP1	2.8 ± 0.7 ^fg^	62.3 ± 7.7 ^ij^	23.3 ± 4.7 ^fg^	12.3 ± 1.6 ^c^	11.6 ± 3.2 ^defg^	112.3 ± 10.4 ^g^
AP2	0.6 ± 0.2 ^g^	62.0 ± 7.5 ^ij^	9.3 ± 0.7 ^ij^	6.8 ± 0.9 ^ef^	12.8 ± 2.0 ^cdef^	91.5 ± 7.8 ^g^
AP3	22.3 ± 4.8 ^c^	54.3 ± 9.6 ^j^	19.6 ± 2.0 ^gh^	9.1 ± 0.4 ^de^	13.5 ± 0.3 ^cdef^	118.8 ± 10.9 ^g^
Mean	8.6 ± 11.9 ^A^	59.5 ± 4.5 ^C^	17.4 ± 7.3 ^A^	9.4 ± 2.8 ^A^	12.6 ± 1.0 ^A^	107.5 ± 14.3 ^B^
*A. horridus*						
H1	35.4 ± 4.5 ^b^	228.6 ± 22.5 ^efgh^	69.4 ± 0.4 ^c^	0.4 ± 0.1 ^h^	13.1 ± 0.8 ^cdef^	346.9 ± 23.0 ^cd^
H2	18.2 ± 4.9 ^cd^	235.0 ± 21.9 ^efgh^	117.8 ± 3.0 ^b^	35.9 ± 3.4 ^a^	14.0 ± 3.1 ^cde^	420.9 ± 23.1 ^c^
H3	n.d.	148.2 ± 17.6 ^hi^	136.2 ± 11.3 ^a^	n.d.	11.9 ± 2.0 ^cdefg^	296.3 ± 21.0 d^ef^
H4	9.7 ± 2.3 ^e^	171.2 ± 11.5 ^gh^	10.4 ± 1.6 ^i^	7.6 ± 0.9 ^ef^	12.9 ± 0.1 ^cdef^	211.8 ± 11.9 ^f^
H5	n.d.	207.3 ± 20.7 ^fgh^	28.5 ± 4.5 ^ef^	1.1 ± 0.3 ^h^	18.4 ± 0.5 ^a^	255.3 ± 21.2 ^ef^
Mean	21.1 ± 13.1 ^A^	198.1 ± 37.4 ^BC^	72.5 ± 54.6 ^A^	11.3 ± 16.8 ^A^	14.1 ± 2.5 ^A^	306.2 ± 81.3 ^AB^
*A. officinalis*						
O1	8.4 ± 0.3 ^ef^	512.2 ± 64.5 ^c^	8.5 ± 2.1 ^ij^	n.d.	9.5 ± 1.7 ^g^	538.6 ± 64.6 ^b^
O2	16.1 ± 3.1 ^d^	636.3 ± 14.3 ^a^	8.7 ± 2.0 ^ij^	n.d.	11.0 ± 1.6 ^fg^	672.1 ± 14.9 ^a^
Mean	12.3 ± 5.4 ^A^	574.3 ± 87.8 ^A^	8.6 ± 0.1 ^A^	-	10.3 ± 1.1 ^A^	605.4 ± 94.4 ^A^

^1^ Data represent means ± SD of samples analyzed in triplicate. ^2^ Differences in phenolic amounts were tested according to one-way ANOVA followed by Duncan’s test. ^3^ In a column, means followed by different lowercase letters are significantly different at *p* < 0.05, and means followed by capital letters represent the ANOVA test effected for mean values of species (*p* < 0.05). ^4^ Quercetin equivalents. ^5^ Rutin equivalents. n.d., not detected.

**Table 3:** Data on sample collection of *Asparagus* species.

Species/Location	Code	Geographical Coordinates	Date
*Asparagus acutifolius* (Raviscanina)			
Arroyo Blanco, Santisteban del Puerto, Jaén	AC1	38.336873, −3.342487	3 April 2022
Mirador de las Latas, Laguna de Fuente Piedra, Málaga	AC2	37.085181, −4.792719	27 March 2022
Umbría de las Yeseras, Navas de San Juan, Jaén	AC3	38.200012, −3.302768	18 April 2022
Rodalquilar, Níjar	AC4	36.849231, −2.043093	7 February 2022
*Asparagus albus* (White *Asparagus*)			
Puerto de Galíz, Cádiz	AL1	36.531646, −5.651272	27 March 2022
Sierra Cabrera, Almería	AL2	37.134984, −1.868005	3 January 2022
Sierra Morena, Jaén	AL3	38.333413, −3.301199	3 March 2022
El Toyo, Almería	AL4	36.847975, −2.332920	8 February 2022
Rodalquilar, Níjar	AL5	36.849231, −2.043093	5 March 2023
*Asparagus aphyllus* (Prickly *Asparagus*)			
Alcalá de los Gazules, Cádiz	AP1	36.493973, −5.692664	27 March 2022
Puerto del Bujeo, Cádiz	AP2	36.071977, −5.516156	26 March 2022
Bujeos Altos, Ubrique, Cádiz	AP3	36.625536, −5.454016	27 March 2022
*Asparagus horridus* (Esparraguera)			
Cabo de Gata, Almería	H1	36.723495, −2.183220	13 March 2022
Calahonda, Granada	H2	36.702389, −3.409915	13 March 2022
Enix, Almería	H3	36.875594, −2.609560	20 March 2022
Las Amoladeras Almería	H4	36.817729, −2.253485	13 March 2022
Rodalquilar, Níjar	H5	36.849231, −2.043093	28 February 2022
*Asparagus officinalis* (cultured *Asparagus*)			
Láchar, Granada	O1	Purchased	4 April 2022
Loja, Granada	O2	Purchased	4 October 2022

## Data Availability

The data presented in this study are available within the article and Supplementary Materials.

## Supplementary Materials

The following supporting information can be downloaded at https://www.mdpi.com/article/10.3390/molecules28155786/s1, Supplementary File S1: Methods, Supplementary Table S1: Selected data on ascorbic acid, phenolics, and antioxidant activity of green *Asparagus* spp. shoots, Supplementary Table S2: Compounds detected in Asparagus samples by LC-MS, Supplementary Table S3: HPLC-DAD and LC-MS parameters used for the analysis of phenolic-enriched extracts of *Asparagus* shoots. References [3,9,10,11,14,19,25,31,37,48,49,50,51,53,54,55,56,57,58,59,60,61,62,63,64,65,66,67,68,69,70,71,72,73,74,75,76,77,78] are cited in the Supplementary Materials.

## Sample Availability

Compound samples are not available from the authors.
