# Supplementary material for: Wild Asparagus Shoots Constitute a Healthy Source of Bioactive Compounds
Source: Molecules. 2023 Jul 31;28(15):5786. doi: 10.3390/molecules28155786 (PMC10421306; doi:10.3390/molecules28155786)
Supplement: Supplementary file 1 [file molecules-28-05786-s001.zip › Supplementary File S1. Methodology.pdf]

Supplementary File S1. Methods

1. Extraction of phenolic compounds from the shoots of *Asparagus* samples

Extraction and analysis of phenolic compounds from *Asparagus* shoots were carried out according to Lyashenko et al. (2021) with some modifications. 1 g of sample was weighed, then 20 mL of ethanol 96% was added, the sample was macerated and centrifuged at 4000 rpm for 10 min; later the sample was passed through filter paper and the supernatant was collected. Then 1 ml of the phenolic extract was taken and filtered again through a 0.22 μm membrane filter before chromatographic analysis.

2. Characterization of phenolic compounds by HPLC-DAD

HPLC analysis of phenols was carried out in Finnigan Surveyor chromatograph equipped with a diode-array detector (DAD) and a reverse-phase C18 column (Hypersil Gold, 250 mm × 4.6 mm i.d., 5 μm particle size) (Thermo Electron, Cambridge, UK). The compounds were separated with a gradient elution using acidified water (1% acetic acid) (A) and acetonitrile (B) as mobile phase at 25 ºC. HPLC grade solvents, Merck and Ultrapure water Milli-Q® were used for HPLC. Solvents were filtered through a 0.45 μm membrane filter (Millipore, Durapore®, Ireland) before use. The gradient started at 100% solvent A (10 min) which was reduced to 98% (10 min), 95% (20 min), 95% (10 min), 90% (10 min), 70% (10 min), 50% (10 min), 100% (15 min), and 100% (5 min). The total running time was 100 min. The flow rate was kept at 0.6 mL/min and the injection volume was 10 μL. Peaks were monitored at 254, 280, and 320 nm, and identified by retention times in comparison with pure standards.

Gallic acid (97.5%, 91215), protocatechuic acid (≥97%, 37580), 4-hydroxybenzoic acid (99%, H20059), DL-*p*-hydroxyphenyllactic acid (≥97%, H3253), 3,4-dihydroxyhydrocinnamic acid (98%, 102601), chlorogenic acid (≥95%, C3878), caffeic acid (≥98%, C0625), vanillic acid (≥97%, 94770), vanillin (99%, V1104), syringic acid (≥95%, S6881), sinapic acid (≥98%, D7927), salicylic acid (≥99%, 247588), *trans*-*p*-coumaric acid (≥98%, 55823), salicylic acid (≥99%, 247588), ferulic acid (99%, 128708), chelidonic acid (98%, 382272), naringenin (≥95%, N5893), rutin (≥94%, R5143), rosmarinic acid (≥98%, R4033), 2-hydroxy-4-methoxybenzoic acid (99%, 173479), quercetin (≥95%, Q4951), luteolin (≥97%, 72511), kaempferol (≥90%, K0133), and all chemicals and solvents were purchased in high purity grade from Merck (Spain).

3. Characterization of phenolic compounds by LC-MS

The chromatographic separation was performed on a Vanquish Flex Quaternary LC equipped with a reverse-phase C18 column (Hypersil Gold, 100 mm × 2.1 mm, 1.9 µm) (Thermo Fisher Scientific, San Jose, CA, USA) at flow rate of 0.2 mL/min. The compounds were separated with gradient elution using acidified water (H_2O containing 0.1% formic acid and 4 mM ammonium formate) (A) and methanol (B) as eluents at room temperature (25 ºC). The step gradient was as follows: 0-1 min 95% of A; then, it was linearly decreased to 70% in 7 min, to 50% in 5 min and remained constant during 5 min. Later, it was decreased to 60% in 5 min, to 100% in 5 min and remained constant during 2 min. Finally, it increased to 95% in 2 min and remained constant during 7 min. The total running time was 39 min. The injection volume was 10 µL.

The LC system was coupled to a hybrid mass spectrometer Q-Orbitrap Thermo Fisher Scientific (Q-Exactive^{TM}, Thermo Fisher Scientific, Bremen, Germany) using electrospray ionization (ESI) (HESI-II, Thermo Fisher Scientific, San Jose, CA, USA) in positive and negative ion mode. ESI parameters were as follows: spray voltage, 4 kV; sheath gas (N_2, 95%), 35 (arbitrary units); auxiliary gas (N_2, 95%), 10 (arbitrary units); S-lens RF level, 50 (arbitrary units); heater temperature, 305 °C, and capillary temperature, 300 °C. The mass spectra were acquired employing four alternating acquisition functions: (1) full MS, ESI+, without fragmentation (the higher collisional dissociation (HCD) collision cell was switched off), mass resolving power = 70,000 Full Width at Half Maximum (FWHM); AGC target = 1e6, scan time = 250 ms; (2) full MS, ESI-, without fragmentation (the higher collisional dissociation (HCD) collision cell was switched off), mass resolving power = 70,000 Full Width at Half Maximum (FWHM); AGC target = 1e6, scan time = 250 ms; (3) data independent analysis (DIA), ESI+, setting higher energy collisional dissociation (HCD) on, and collision energy = 30 eV, mass resolving power = 35,000 FWHM, scan time = 125 ms; (4) DIA, ESI- (setting HCD on, and collision energy = 30 eV), mass resolving power = 35,000 FWHM, scan time = 125 ms. The mass range in the full scan MS experiments was set to *m/z* 50-750. LC chromatograms were acquired using the external calibration mode and they were processed using Xcalibur^{TM} version 3.0, with Qualbrowser and Trace Finder 4.0 (Thermo Fisher Scientific, Les Ulis, France). Unknown analysis was carried out with Compound Discoverer^{TM} version 2.1 (Thermo Scientific, Les Ulis, France).

4. Accuracy and validity of the HPLC-DAD methodology

HPLC-DAD analyses were performed using the experimental set-up previously described (Lahlou et al., 2022). Precision/injection repeatability test (expressed as relative standard deviation, %) was performed by five replicated injections of the standard mixture of phenolic acids (PAs) at a concentration of 1.25, 2.5, 5.0, 10.0 and 50.0 ppm. The accuracy was also evaluated by the standard addition procedure (% of recovery) with three addition levels (30% of the expected values, in duplicate). Standard mixture was added to the samples, and all the extraction procedures were carried out. Recovery rates were calculated by the following equation:

$R\% = [(Cs-Cp)/Ca]\cdot$

where R (%) is the percent recovery of added standard; Cs the compound content in spiked sample; Cp the compound content in sample; and Ca the compound standard added. To determine the limits of detection (LOD) and quantification (LOQ) of the assays, pure phenolic compounds were diluted in the 0.001-20 mg/mL range in methanol in triplicate and quantified by HPLC-DAD. Negative controls made without phenolics addition were also analyzed. The LOD was defined as the minimum concentration at which distinct peaks could be detected above the baseline noise. The LOQ was defined as the lowest concentration of compounds that could be quantified with an accuracy and precision within 15%.

5. Cell assay

Antiproliferative activity of hydroalcoholic ethanol 96% extracts from *Asparagus* shoots was assayed on the HT-29 human colon cancer cell line as described by Ramos-Bueno *et al.* (2017). Cell cultures were supplied by the Technical Instrumentation Service of University of Granada (Granada, Spain). After being checked for the absence of *Mycoplasma* and bacteria, cells were grown at 37 ºC and 5% CO_2 humidified atmosphere in medium RPMI-1640 supplemented with 5% fetal bovine serum, 2 mM L-Glutamine, 1 mM Sodium pyruvate, 0.125 mg/mL Amphotericin B and 100 mg/mL Penicillin-Streptomycin. All cultures were plated in 25 cm^2 plastic tissue culture flasks (Sarstedt, USA). All culture media and reagents were purchased from Sigma-Aldrich (St. Louis, MO, USA). Cell culture and cell assay, that is, the 3-(4,5-dimethylthiazol-2-yl)-2,5-diphenyltetrazolium bromide (MTT) test were accomplished as previous described (Ramos-Bueno *et al.*, 2016).

In the MTT assay, cells were divided into 96-well microtiter plates, adjusted of 1×10^4 cell/well and cultivated in medium at 37 ºC, 5% CO_2 prior to adding the different

extracts dissolved in medium. The phenolics-containing extracts of *Asparagus* shoots were supplied to cells dissolved in ethanol 96% and then in the culture medium at designed concentrations (0-1000 μg/mL). 48 and 72 h later, 5 mg/mL of an MTT solution was added to the culture medium to determine the viability of cells. The absorbance was recorded at 570 nm on an enzyme-linked immunosorbent assay (ELISA) plate reader (Thermo Electron Corporation, Sant Cugat del Valles, Barcelona, Spain) (Ramos-Bueno et al., 2016). The formazan crystals produced were solubilized using 100 μL dimethyl sulfoxide (DMSO). Cells without phenolic extracts were considered as negative controls. Cell survival in exposed cultures relative to unexposed cultures (negative controls) was calculated, and the number of viable cells was calculated using the following equation:

Percentage of viable cell (%) = (Absorbance of treated cells/Absorbance of untreated cells) × 100%.

The concentrations causing 50% cell growth inhibition (GI_{50}) were calculated from the growth curves. Phenolic extracts, pure phenolics and controls were evaluated in three independent assays. Values presented are mean ± standard error of the mean.

6. Determination of the total phenolic content

Total phenolics content (TPC) was measured using the Folin-Ciocalteu assay developed by Singleton *et al.* (1999) with minor modifications. Briefly, 10 μL of *Asparagus* phenolic extracts (preparation of the phenolic extract is fully detailed in Supplemental File 1-1), 0.79 mL of MiliQ water, and 50 μL of Folin-Ciocalteu reagent were mixed, vortex and allowed to stand for 5 min at room temperature. Next, 150 μL of a 20% sodium carbonate solution were added and vortex. After incubation at room temperature for 2 h in darkness, the absorbance of the mixture was read at 765 nm on a UV-VIS spectrophotometer (Helios, Thermo Spectronic, Cambridge, UK) against the blank, which was prepared as described above but without standard solutions. The results were expressed as mg of gallic acid equivalents (GAE) per 100 g of sample using a standard curve of gallic acid (ranging from 50 to 900 μg/mL). Determinations were done in triplicate.

7. Determination of total flavonoid content

Total flavonoid content (TFC) of the phenolic extract of *Asparagus* shoots was determined according to Zou *et al.* (2004) with some modifications. Briefly, 0.5 mL of the phenolic extracts was taken and 150 μL of 5% $NaNO_2$ solution was added. After 5 min, 150 μL of 10% $AlCl_3$ solution was added to the mixture, which was kept at room

temperature for 5 more minutes, followed by the addition of 0.7 ml of 1M NaOH. The resulting solution was mixed well and immediately, the absorbance was measured at 510 nm on a UV-VIS spectrophotometer (Helios, Thermo Spectronic, Cambridge, UK) against a blank, which was prepared as described above but without standard solutions. The results were expressed as mg of quercetin equivalents (QE) per 100 g of sample using a standard curve of quercetin (ranging from 10 to 500 μg/mL). Determinations were done in triplicate.

8. Extraction and quantification of vitamin C

Vitamin C (L-ascorbic acid) content was determined according to Volden et al. (2009) with minor modifications. Briefly, 1 g of fresh *Asparagus* shoots sample was extracted with 20 mL of 1% (w/v) oxalic acid. The mixture was centrifuged at 4000 rpm for 10 min, later the sample was passed through filter paper and the supernatant was collected. Then, 1 ml of the vitamin C extract was taken and filtered again through a 0.22 µm Millipore before chromatographic analysis.

HPLC analysis of vitamin C was carried out in Finnigan Surveyor chromatograph equipped with a diode-array detector (DAD) and a reverse-phase C18 column (Hypersil Gold, 250 mm × 4.6 mm i.d., 5 μm particle size) (Thermo Electron, Cambridge, UK). The mobile phase consisted of 5% (v/v) methanol:water (0.1% oxalic acid in water) in isocratic mode was used. The wavelength detection was set at 254 nm. The flow rate of the mobile phase was 0.4 mL/min and the volume injected was 10 µL. The total running time was 15 min. Ascorbic acid was quantified by external calibration and results were recorded as mg/100 g fresh weight. All data are presented as mean ± standard deviation of triplicate analysis.

2.9. Determination of the antioxidant activity

Methanolic extraction was carried out according to the methodology described by Forbes-Hernández et al. (2017) with some modifications. One g of sample was weighed, then 15 mL of an ethanol 96% was added, the sample was macerated and centrifuged at 3500 rpm for 5 min; later the sample was passed through filter paper and the supernatant was collected. The antioxidant activity using the $ABTS^{\bullet+}$ method was determined using a solution of $ABTS^{\bullet+}$ radical (2,2'-azinobis (3-Ethylbenzothiazoline-6-sulfonic acid) in ethanol (2.45 mM). Subsequently, 1800 μL aliquot was removed from the $ABTS^{\bullet+}$ solution and 200 μL of the methanolic sample extract was added, mixed at 3000 rpm for 30 s in a vortex, and placed in darkness for 7 min. The absorbance was measured at 734 nm on a UV-VIS spectrophotometer (Helios, Thermo

Spectronic, Cambridge, UK) against a blank, which was prepared as described above but without standard solutions.

The DPPH method was carried out according to Skenderidis's et al. (2018) methodology with some modifications. A DPPH solution (2,2-diphenyl-1-picrylhydrazyl) was prepared in methanol (0.25 mM). From the DPPH solution, 1800 µL was taken and 200 µL of the methanolic sample extract was added, vortexed at 3000 rpm for 30 s, and allowed to react in the dark for 30 min. The absorbance of the solution was measured at 517 nm on a UV-VIS spectrophotometer (Helios, Thermo Spectronic, Cambridge, UK) against a blank, which was prepared as described above but without standard solutions. The values of $ABTS^{\bullet+}$ and DPPH were expressed as Trolox equivalent mg/100 g dry weight (mg TE/100 g dw).

References

Forbes-Hernández, T. Y., Gasparrini, M., Afrin, S., Cianciosi, D., González-Paramás, A. M., Santos-Buelga, C., ... Bompadre, S. (2017). Strawberry (cv. Romina) methanolic extract and anthocyanin-enriched fraction improve lipid profile and antioxidant status in HepG2 cells. *International Journal of Molecular Sciences, 18,* 1149. https://doi.org/10.3390/ijms18061149.

Lahlou, A., Chileh-Chelh, T., Lyashenko, S., Rincón-Cervera, M. Á., Rodríguez-García, I., López-Ruiz, R., ... Guil-Guerrero, J. L. (2022). Arecaceae fruits: Fatty acids, phenolic compounds and *in vitro* antitumor activity. *Food Bioscience, 50,* 102181. https://doi.org/10.1016/j.fbio.2022.102181.

Lyashenko, S., Fabrikov, D., González-Fernández, M. J., Gómez-Mercado, F., Ruiz, R. L., Fedorov, A., Guil-Guerrero, J. L. (2021). Phenolic composition and *in vitro* antiproliferative activity of *Borago* spp. seed extracts on HT-29 cancer cells. *Food Bioscience, 42,* 101043. https://doi.org/10.1016/j.fbio.2021.101043.

Ramos-Bueno, R. P., González-Fernández, M. J., Guil-Guerrero, J. L. (2016). Various acylglycerols from common oils exert different antitumor activities on colorectal cancer cells. *Nutrition and Cancer, 68,* 518-529. https://doi.org/10.1080/01635581.2016.1152382.

Ramos-Bueno, R. P., Romero-González, R., González-Fernández, M. J., Guil-Guerrero, J. L. (2017). Phytochemical composition and *in vitro* anti-tumour activities of selected tomato varieties. *Journal of the Science of Food and Agriculture, 97,* 488-496. https://doi.org/10.1002/jsfa.7750.

Singleton, V. L., Orthofer, R., Lamuela-Raventós, R. M. (1999). Analysis of total phenols and other oxidation substrates and antioxidants by means of Folin-Ciocalteu reagent. *Methods in Enzymology*, 2*99*, 152-178.
https://doi.org/10.1016/S0076-6879(99)99017-1.
Skenderidis, P., Kerasioti, E., Karkanta, E., Stagos, D., Kouretas, D., Petrotos, K., ... Tsakalof, A. (2018). Assessment of the antioxidant and antimutagenic activity of extracts from goji berry of Greek cultivation. *Toxicology Reports*, *5*, 251-257.
https://doi.org/10.1016/j.toxrep.2018.02.001.
Volden, J., Bengtsson, G. B., Wicklund, T. (2009). Glucosinolates, L-ascorbic acid, total phenols, anthocyanins, antioxidant capacities and colour in cauliflower (*Brassica oleracea* L. ssp. *botrytis*); effects of long-term freezer storage. *Food Chemistry*, *112*, 967-976. https://doi.org/10.1016/j.foodchem.2008.07.018.
Zou Y., Lu Y., Wei, D. (2004). Antioxidant activity of flavonoid-rich extracts of *Hypericum perforatum* L. *in vitro*. *Journal of Agricultural and Food Chemistry*, *52*, 5032-5039. https://doi.org/10.1021/jf049571r.
