# Supplementary material for: Wild Asparagus Shoots Constitute a Healthy Source of Bioactive Compounds
Source: Molecules. 2023 Jul 31;28(15):5786. doi: 10.3390/molecules28155786 (PMC10421306; doi:10.3390/molecules28155786)
Supplement: Supplementary file 1 [file molecules-28-05786-s001.zip › Supplementary Table S1. Review.pdf]

Supplementary Table S1. Selected data on ascorbic acid, phenolics, and antioxidant activity of green *Asparagus* spp. shoots

Codes/Species	Vitamin C mg/100 g fw	Main phenolics	Total phenolics[c]	Antioxidant activity	References
A. acutifolius					
RAC1	15.6	CfA 24.4; I 249; K 290; My 63; M3O 235.4; PrA 19.1; Q 1240; QG 186 mg/kg fw	TPA 43; TFC 2263 mg/kg fw	-	Salvatore et al., 2005
RAC2	117	CiA 0.1; Med 1.2; Sir 0.5; SA 0.6; BA 1.2; FA 0.5; CouA 0.6; SyrA 1.0; 3,4-diOMeP 1.5; *p*HBA 1.2; *p*HPAA 1.0; C 1.3; PrA 1.5 mg/100 g fw	TPC 41.97 mg/100 g fw	-	Ferrara et al., 2011
RAC3	-	Nic 2.2; R 26.3; SAP 5.3; Nar 1.6 mg/100 g fw	TPA 35.4 mg/100 g fw TFC 30.9 mg/100 g fw	-	Barros et al., 2011
RAC4	21.9	-	-	-	Martins et al., 2011
RAC5	-	BA 5; CfA 2; ClA 4; CouA 2; Ct 1; FA 3; GA 26; PrA 4; QG 25; R 28; % of TP	TPA 2289 mg GAE/100 g fw	-	Di Maro et al., 2013
RAC6 Three extraction systems	-	Bound P: Rut 0.1-3.0; Nic 0.0-0.2; Nar 0.2-2.1 g/kg dw Free P: Rut 0.1-0.9; Nic 0.0-0.1; Nar 0.1-0.8 g/kg dw	TPA 200-800 mg GAE/100 g dw; TFC 140-160 mg CE/100 g dw	ABTS 0.1-2.1 mg TE/100 g dw DPPH 0.3-1.1 mg TE/100 g dw	Poljuha et al., 2015
RAC7	-	-	TPA 620 mg GAE/100 g fw	-	Palfi et al., 2017
RAC8	-	ClA 55; CfA+CfA der 30; ChA 65; CFQA 25; Rut 299; Nic 18; Nar 215 mg/100 g dw	TPC 1200 mg CAE/100 g dw	-	Sergio et al., 2020
RAC9 var. wild green	-	ClA 66.76; ChA 82.08; Rut 80.41; Nar 95.69 mg/100 g dw	TFC 850 mg CE/100 g dw	ABTS 0.95 mg TE/100 g dw	Sergio et al., 2021
RAC9 var. wild violet	-	ClA 70.16; ChA 30.94; Rut 203.03; Nar 154.16 mg/100 g dw	TP 1000 mg CE/100 g dw	ABTS 0.75 mg TE/100 g dw	Sergio et al., 2021
RAC10	-	PrA 26; *p*HBA 15; VA 11; CfA 297; *p*HBA 10; SyrA 1; VN 12; CouA 7; FA 28; SAP 9; R 316; Nar 154; NG 61; K 5 mg/kg dw	TPA 415; TFC 537 mg/kg dw		Hamdi et al., 2021
A. horridus					
RAH1	-	CFGI 3.9; CFGII 1.1; FG 1.1; dFGI 7.1; dFGII 2.0; FH 1.5; IH 2.7; Nar 1.0; KH 4.9; QOD 1.2; Rut 1.1 mg/100 g dw	TPA 18.7 mg/100 g dw TFC 10.9 mg/100 g dw.		Adouni et al., 2018
A. officinalis					
RAO1	23 mg/g dw			ABTS 65.7 µmol/g dw	Nindo et al., 2003
RAO2		Rut 30-60 mg/100 g fw			Wang et al., 2003

RAO3		FA 40-182; FAd 0-580; CouA 92-439; *p*HBA 1-2; VA 1-5; *p*HBD 1-7; VN 5-41 mg/kg fw			Rodríguez et al., 2005
RAO4			TPC 4.9-5.0 mg CE/g dw<br>TFC 1.5-4.7 mg RE/g dw	DPPH 10.9-17.4 mM TE/kg dw<br>ABTS 26.2-27.1 mM TE/kg dw	Sun et al., 2007
RAO5	23	CiA 0.1; Med 0.5; Sir 0.1; SA 0.2; BA 0.2; FA 0.1; CouA 0.4; SyrA 0.6; 3,4-diOMeP 0.4; *p*HBA 0.6; pHPAA 0.3; C 0.6; PrA 0.3 mg/100 g fw	27.62 mg/100 g fw		Ferrara et al., 2011
RAO6	12		TPA 1.9-2.8 mg GAE/100 g fw<br>TFC 0.7-2.2 RE/100 g fw	DPPH 0.63-2.04 µmol TE/kg fw	Wei et al., 2011
RAO7	-	Rut 1900-6700 mg/100 dw	-	-	Motoki et al., 2012
RAO8<br>CO_2-SFE extract	-	Rut 67; QG 10; 3FerQA 9; Nar 5; Nic 2; CfAH 2; ClA 2; Q 1 % of TPC	TPC 280 mg/100 g dw	-	Solana et al., 2015
RAO9	-	-	TPA 452 mg GAE/100 g fw	-	Palfi et al., 2017
RAO10<br>(5 cultivars)	2.4-5.9	CfA 0.0-1.3; ClA 0.0-9.3; CouA 0.0-2.3; FA 0.0-5.8; Ki 1.5-2.2; *p*HBA 0.2-0.3; Q 1.3-2.3; R 2.9-23.8 mg/kg fw	TPA 12-43 mg GAE/kg fw<br>TF7-28 mg RE/kg fw	-	Chen et al., 2017
RAO11<br>(8 cultivars)	-	*p*CouH 1.5-5.2; oCouQA 1.6-7.0; 3FerQA 1.8-7.1; 4FerQA 2.0-3.6; CFQA 33.0-57.2; QRH 7.2-96.4; Rut 94.0-129.3; QG 1.8-6.8; Nar 2.4-7.1; KH 1.2-16.8; Nic 2.0-7.5; IH 2.4-3.4; AR 1.0-10.4; CyRH 0.1-7; CyRut 1.1-58.5; PRut 0.1-1.0 mg/kg dw	TPA 445-685 mg GAE/kg fw	-	Slatnar et al., 2017
RAO12<br>(MeOH extract), several agronomic conditions	7.14-20.40 mg/g dw		TPA 11.22-387 mg GAE/g dw<br>TFC ~5-9 mg CE/g dw	ABTS 3.14-10.77 mM TE/kg dw	Ku et al., 2018
RAO13<br>(4 drying methods)	-	Free P: GA 46.4-151-4; Rut 101.4-117.9; CiA 44.3-159.4; FA 160.9-244.8; Q 75.4-118.7 µg/g dw<br>Bound P: GA 18.2-19.9; Rut 23.1-39.3; CiA 52.9-154.9; FA 20.6-66.3; Q 0.0-34.3; K 21.1-23.1 µg/g dw	TPC free: 605.6-791.8<br>TPC bound: 527.4-631.3 µg GAE/g dw<br>TFC: 592.7-837.6 µg CE/g dw	-	Yu et al., 2019
RAO14<br>(5 cultivars)	-	Rut 550-1200 mg/100 g dw	TPA: 936-720 CAE/100 g dw	ABTS 53.7-93.2 mg TE/100 g dw	Sergio et al., 2019
RAO15<br>(three genotypes and 5 growing conditions)	-	GA 30.06-341.79; PrA 0.00-22.19; *p*HBA 0.00-64.32; VA 0.00- 179.72; CfA 0.00- 32.50; CouA 0.00-178.17; FA 56.13-153.41; SNA 16.32-85.81; Rut 31.85- 16318.67; Q3G 0.00- 97.08; Hy 0.00-197.73; K3OG 0.00-57.96; Q 0.00-57.96 µg/100 g dw			Kobus-Cisowska et al., 2019

RAO16	-	CFQA 0.68; CafAH 0.35; VC 2.05; Rut 15.01; Nic 0.36; Nar 0.53 mg/kg dw	TPC 5.57-6.69 mg/g dw	Noperi-Mosqueda et al., 2020
RAO17<br>23 varieties checked	-	Rut 33.8-1624.8; Nic 17.3-214.7 mg/kg dw	TPC 873.7-2,043.8 mg GAE/g dw<br>TFC 18.5-190.1 mg QE/ g dw	Eum et al., 2020
RAO18<br>3 extracting solvents checked	-	ClA 1.21-5.51; FA 1.25-4.81; Rut 30.74-92.63; EA 1.36-4.74 µg/g fw		Ayhan & Rosenberg, 2021
RAO19<br>2 cultivation systems at 2 stages of development	-	CfA 4.0-7.1; CouA 0.65-0.81; FA 1.5-2.4; Q 3.5-6.6; SNA 11.3-14.4; Rut 234.6-442.9 mg/100 g dw	TPA 37-40 mg GAE/g dw<br>TFC 27-29 mg QE/g dw	Cao et al., 2022
RAO20	-	QG 0.36; IH 0.27; Rut 8.10; Nic 0.07; Nar 0.06 mg/100 g dw	TFC 8.87 mg/100 g dw	Redondo-Cuenca et al., 2023

Abbreviations: CAE: caffeic acid equivalents; CE: catechin equivalents; GAE: gallic acid equivalents; dw: dry weight; fw: fresh weight; RE: rutin equivalents; TE: trolox equivalent; TFC: total flavonoids content; TPC: total phenolics content; TPA: total phenolic acids.

Phenolics: AR: Apigenin rhamnoside; BA: benzoic acid; C: cathecol; Ct: catechin; ChA: chicoric acid; CiA: cinnamic acid; ClA: Chlorogenic acid; CouA: coumaric acid; CfA: Caffeic acid; CFQA: Caffeoyl-quinic acid; CfAH: Caffeic acid hexose; CFGI: Coumaroylferuloyl glycerol I; CFGII: Coumaroylferuloyl glycerol II; CyRH: Cyanidin rhamnosyl hexosides; CyRut: Cyanidin rutinoside; 3,4-diOMeP: 3,4-dimethoxyphenol; EA: ellagic acid; FA: ferulic acid; FAd: ferulic acid dimers; FG: Feruloyl glycerol; dFGI: Diferuloyl glycerol I; dFGII Diferuloyl glycerol II; 3FerQA: 3-Feruloylquinic acid; 4FerQA: 4-Feruloylquinic acid; FH: Feruloylhexoside; GA: gallic acid; Hy: Hyperoside (quercetin 3-*O*-galactoside); I: Isorhamnetin; IH: Isorhamnetin hexosides; K: Kaempferol; Ki: Kaemferide; KH: Kaempferol hexosides; K3OG: Kaempferol 3-*O*-glucoside (astragalin); Nar: narcisin (isorhamnetin-3-O-rutinoside); Nic: nicotiflorin (kaempferol 3-*O*-rutinoside); Med: mediaresinol; My: myricetin; M3O: myricetin-3-*O*-glucoside; NG: naringenin; oCouQA: *o*-Coumaroylquinic acid; PRut: Peonidin rutinoside; *p*HBA: *p*-hydroxybenzaldehyde; *p*CouH: *p*-Coumaric acid hexoside; *p*HBA: *p*-hydroxybenzoic acid; *p*HBD: *p*-hydroxybenzaldehyde; *p*HPAA: *p*-hydroxyphenyl acetic acid; PrA: prothocathecuic acid; Q: quercetin; QG: quercetin glycosides; Q3G: Quercetin-3-glucoside (Isoquercetin); QOD: quercetin-*O*-dideoxyhexosylhexoside; QRH: Quercetin rhamnosyl hexosides; Rut: rutin (quercetin 3-*O*-rutinoside); SA: salicylic acid; SNA: sinapic acid; SNAP: sinapic acid pentoside; Sir: siringaresinol; SyrA: syringic acid; VA: vanillic acid; VC: vicenin 2; VN: vanillin.

References:

Adouni, K., Chahdoura, H., Mosbah, H., Santos-Buelga, C., González-Paramás, A. M., Cuidad-Mulero, M., Fernandes, Â., Calhelha, R. C., Morales, P., Flamini, G., Ferreira, I. C. F. R., Achour, L. (2018). Revalorization of wild *Asparagus stipularis* Forssk. as a traditional vegetable with nutritional and functional properties. *Food & Function*, *9*, 1578-1586. https://doi.org/10.1039/C7FO01687E.

Ayhan, N. K., & Rosenberg, E. (2021). Development of comprehensive liquid chromatography with diode array and mass spectrometric detection for the characterization of (poly-) phenolic and flavonoid compounds and application to asparagus. *Food Chemistry*, *354*, 129518. https://doi.org/10.1016/ j.foodchem.2021.129518.

Barros, L., Dueñas, M., Ferreira, I. C., Carvalho, A. M., Santos-Buelga, C. (2011). Use of HPLC–DAD–ESI/MS to profile phenolic compounds in edible wild greens from Portugal. *Food Chemistry*, *127*, 169-173. https://doi.org/10.1016/j.foodchem.2011.01.009.

Cao, T. L., Cho, N., Lee, T. H., Ahn, S. J., Lee, D. J., & Ku, Y. G. (2022). Bioactive substances, antioxidant enzymes, and anti-cancer activity of asparagus 'atlas' grown in an open field and rain-shelter house system. *Horticulture, Environment, and Biotechnology*, *63*, 809-821. https://doi.org/10.1007/s13580-022-00470-2.

Chen, X. H., Ma, L. H., Dong, Y. W., Song, H., Pu, Y., Zhou, Q. Y. (2017). Evaluation of the differences in phenolic compounds and antioxidant activities of five green asparagus (*Asparagus officinalis* L.) cultivars. *Quality Assurance and Safety of Crops & Foods*, *9*, 479-487. https://doi.org/10.3920/QAS2017.1082.

Di Maro, A., Pacifico, S., Fiorentino, A., Galasso, S., Gallicchio, M., Guida, V., Severino, V., Monaco, P., Parente, A. (2013). Raviscanina wild asparagus (*Asparagus acutifolius* L.): A nutritionally valuable crop with antioxidant and antiproliferative properties. *Food Research International*, *53*, 180-188.
https://doi.org/10.1016/j.foodres.2013.04.026.

Eum H. L., Yi T. G., Hong S. J., Park N. I. (2020). Variations of bioactive compound contents and antioxidant capacity of asparagus seedlings in 23 varieties. *Horticultural Science and Technology, 38*, 291-302. https://doi.org/10.7235/HORT.20200028.

Ferrara, L., Dosi, R., Di Maro, A., Guida, V., Cefarelli, G., Pacifico, S., Mastellone, C., Fiorentino, A., Rosati, A., Parente, A. (2011). Nutritional values, metabolic profile and radical scavenging capacities of wild asparagus (*A. acutifolius* L.). *Journal of Food Composition and Analysis, 24*, 326-333. https://doi.org/10.1016/j.jfca.2010.10.008.

Hamdi, A., Jaramillo-Carmona, S., Rodríguez-Arcos, R., Jiménez-Araujo, A., Lachaal, M., Karray-Bouraoui, N., Guillén-Bejarano, R. (2021). Phytochemical Characterization and Bioactivity of *Asparagus acutifolius*: A Focus on Antioxidant, Cytotoxic, Lipase Inhibitory and Antimicrobial Activities. *Molecules*, *26*, 3328.
https://doi.org/10.3390/molecules26113328

Kobus-Cisowska, J., Szymanowska, D., Szczepaniak, O. M., Gramza-Michałowska, A., Kmiecik, D., Kulczyński, B., Szulc, P., Górnaś, P. (2019). Composition of polyphenols of asparagus spears (*Asparagus officinalis*) and their antioxidant potential. *Ciência Rural, 49*. https://doi.org/10.1590/0103-8478cr20180863.

Ku, Y. G., Kang, D. H., Lee, C. K., Lee, S. Y., Ryu, C., Kim, D. E., Polovka, M., Namieśnik, J., Gorinstein, S. (2018). Influence of different cultivation systems on bioactivity of asparagus. *Food Chemistry*, *244*, 349-358. https://doi.org/10.1016/j.foodchem.2017.10.044.

Lee, E. J., Yoo, K. S., Patil, B. S. (2010). Development of a rapid HPLC-UV method for simultaneous quantification of protodioscin and rutin in white and green asparagus spears. *Journal of Food Science*, *75*, C703-C709. https://doi.org/10.1111/j.1750-3841.2010.01824.x.

Martins, D., Barros, L., Carvalho, A. M., Ferreira, I. C. (2011). Nutritional and in vitro antioxidant properties of edible wild greens in Iberian Peninsula traditional diet. *Food Chemistry*, *125*, 488-494. https://doi.org/10.1016/j.foodchem.2010.09.038.

Motoki, S., Kitazawa, H., Maeda, T., Suzuki, T., Chiji, H., Nishihara, E., Shinohara, Y. (2012). Effects of various asparagus production methods on rutin and protodioscin contents in spears and cladophylls. Bioscience, Biotechnology, and Biochemistry, *76*, 1047-1050. https://doi.org/10.1271/bbb.120143.

Nindo, C., Sun, T., Wang, S. W., Tang, J., Powers, J. R. (2003). Evaluation of drying technologies for retention of physical quality and antioxidants in asparagus (*Asparagus officinalis* L.). *LWT-Food Science and Technology*, *36*, 507-516. https://doi.org/10.1016/S0023-6438(03)00046-X.

Noperi-Mosqueda, L. C., López-Moreno, F. J., Navarro-León, E., Sánchez, E., Blasco, B., Moreno, D. A., Soriano, T., Ruiz, J. M. (2020). Effects of asparagus decline on nutrients and phenolic compounds, spear quality, and allelopathy. *Scientia Horticulturae*, *261*, 109029. https://doi.org/10.1016/j.scienta.2019.109029.

Palfi, M., Jurković, Z., Ćosić, J., Tomić Obrdalj, H., Jurković, V., Knežević, N., Vrandečić, K. (2017). Total phenol content and antioxidant activity of wild and cultivated asparagus in Croatia. *Poljoprivreda*, *23*, 56-62. https://doi.org/10.18047/poljo.23.1.9.

Poljuha, D., Šola, I., Bilić, J., Dudaš, S., Bilušić, T., Markić, J., Rusak, G. (2015). Phenolic composition, antioxidant capacity, energy content and gastrointestinal stability of Croatian wild edible plants. *European Food Research and Technology*, *241*, 573-585. https://doi.org/10.1007/s00217-015-2486-y.

Redondo-Cuenca, A., García-Alonso, A., Rodríguez-Arcos, R., Castro, I., Alba, C., Rodríguez, J. M., Goñi, I. (2023). Nutritional composition of green asparagus (*Asparagus officinalis* L.), edible part and by-products, and assessment of their effect on the growth of human gut-associated bacteria. *Food Research International*, *163*, 112284. https://doi.org/10.1016/j.foodres.2022.112284.

Rodríguez, R., Jaramillo, S., Guillén, R., Jiménez, A., Fernández-Bolaños, J., Heredia, A. (2005). Cell wall phenolics of white and green asparagus. *Journal of the Science of Food and Agriculture*, *85*, 971-978. https://doi.org/10.1002/jsfa.2053.

Salvatore, S., Pellegrini, N., Brenna, O. V., Del Rio, D., Frasca, G., Brighenti, F., Tumino, R. (2005). Antioxidant characterization of some Sicilian edible wild greens. *Journal of Agricultural and Food Chemistry*, *53*, 9465-9471. https://doi.org/10.1021/jf051806r.

Sergio, L., Gonnella, M., Renna, M., Linsalata, V., Gatto, M. A., Boari, F., Di Venere, D. (2019). Biochemical traits of asparagus cultivars and quality changes in two differently coloured genotypes during cold storage. *LWT*, *101*, 427-434. https://doi.org/10.1016/j.lwt.2018.11.054.

Sergio, L., Boari, F., Pieralice, M., Linsalata, V., Cantore, V., Di Venere, D. (2020). Bioactive phenolics and antioxidant capacity of some wild edible greens as affected by different cooking treatments. *Foods*, *9*, 1320. https://doi.org/10.3390/foods9091320.

Sergio, L., Boari, F., Di Venere, D., Gonnella, M., Cantore, V., Renna, M. (2021). Quality evaluation of wild and cultivated asparagus: A comparison between raw and steamed spears. *Agriculture*, *11*, 1213. https://doi.org/10.3390/agriculture11121213.

Slatnar, A., Petkovsek, M. M., Stampar, F., Veberic, R., Horvat, J., Jakse, M., Sircelj, H. (2018). Game of tones: Sugars, organic acids, and phenolics in green and purple asparagus (*Asparagus officinalis* L.) cultivars. *Turkish Journal of Agriculture and Forestry*, *42*, 55-66. https://doi.org/10.3906/tar-1707-44.

Solana, M., Boschiero, I., Dall'Acqua, S., Bertucco, A. (2015). A comparison between supercritical fluid and pressurized liquid extraction methods for obtaining phenolic compounds from *Asparagus officinalis* L. *The Journal of Supercritical Fluids*, *100*, 201-208. https://doi.org/10.1016/j.supflu.2015.02.014.

Sun, T., Powers, J. R., Tang, J. (2007). Evaluation of the antioxidant activity of *asparagus*, broccoli and their juices. *Food Chemistry*, *105*, 101-106. https://doi.org/10.1016/j.foodchem.2007.03.048.

Wang, M., Tadmor, Y., Wu, Q. L., Chin, C. K., Garrison, S. A., Simon, J. E. (2003). Quantification of protodioscin and rutin in asparagus shoots by LC/MS and HPLC methods. *Journal of Agricultural and Food Chemistry*, *51*, 6132-6136. https://doi.org/10.1021/jf0344587.

Wei, Y., Liu, Z., Su, Y., Liu, D., Ye, X. (2011). Effect of salicylic acid treatment on postharvest quality, antioxidant activities, and free polyamines of asparagus. *Journal of Food Science*, *76*, S126-S132. https://doi.org/10.1111/j.1750-3841.2010.01987.x.

Yu, Q., Li, J., Fan, L. (2019). Effect of drying methods on the microstructure, bioactivity substances, and antityrosinase activity of asparagus stems. *Journal of Agricultural and Food Chemistry*, *67*, 1537-1545. https://doi.org/10.1021/acs.jafc.8b05993
