# Supplementary material for: Wild Asparagus Shoots Constitute a Healthy Source of Bioactive Compounds
Source: Molecules. 2023 Jul 31;28(15):5786. doi: 10.3390/molecules28155786 (PMC10421306; doi:10.3390/molecules28155786)
Supplement: Supplementary file 1 [file molecules-28-05786-s001.zip › Supplementary Table S2 . Phenolics compounds LC_MS.pdf]

Supplementary Table S2. Compounds detected in *Asparagus* samples by LC-MS

	Phenolic compounds										
Species/codes	Chelidonic acid	Caffeic acid	*Trans*-ferulic acid	Syringic acid	Sinapic acid	Naringenin	Quercetin	(-)-Gallocatechin	Quercetin 3'-methyl ether (Isorhamnetin)	Myricetin	*p*-coumaric acid hexoside
A. acutifolius											
AC1	X						X			X	X
AC2	X		X				X			X	X
AC3	X						X			X	X
AC4	X						X			X	X
A. albus											
AL1	X		X	X		X	X			X	X
AL2	X		X	X		X	X			X	X
AL3	X		X	X		X	X				X
AL4	X		X	X			X				X
AL5	X		X	X			X				X
A. aphyllus											
AP1	X						X	X		X	X
AP2	X		X		X	X	X	X		X	X
AP3	X		X			X	X			X	X
A. horridus											
H1	X						X	X		X	X
H2	X						X		X	X	X
H3	X		X			X	X	X	X	X	X
H4	X	X					X		X		X
H5	X						X	X			X
A. officinalis											
O1	X		X				X				X
O2	X	X	X		X		X				X

Supplemental Table 4. Compounds detected in *Asparagus* samples by LC-MS (continued)

Species/codes	Phenolic compounds									
	5-*O*-*p*-coumaroyl quinic acid	Chlorogenic acid	(-)-Epicatechin gallate	Kaempferol-3-*O*-glucoside (Astragalin)	Quercetin 3-*O*-glucoside (Isoquercetin)	Isorhamnetin-3-*O*-glucoside (Asterin)	Kaempferol-3-*O*-rutinoside (Nicotiflorin)	Pelargonidin 3-*O*-diglucoside	Quercetin-3-O-rutinoside (Rutin)	Isorhamnetin-3-*O*-rutinoside (Narcissin)
A. acutifolius										
AC1		X					X	X	X	X
AC2		X				X	X	X	X	X
AC3		X				X	X	X	X	X
AC4				X		X	X	X	X	X
A. albus										
AL1	X	X	X			X	X	X	X	X
AL2	X	X	X			X	X	X	X	X
AL3	X	X	X				X	X	X	X
AL4	X	X	X				X	X	X	X
AL5	X	X	X				X	X	X	X
A. aphyllus										
AP1		X				X	X		X	X
AP2		X				X	X	X	X	X
AP3		X				X	X	X	X	X
A. horridus										
H1						X	X		X	X
H2						X	X		X	X
H3							X		X	X
H4						X	X		X	X
H5							X		X	X
A. officinalis										
O1		X			X	X	X	X	X	X
O2		X			X	X	X	X	X	X
