# Supplementary material for: Wild Asparagus Shoots Constitute a Healthy Source of Bioactive Compounds
Source: Molecules. 2023 Jul 31;28(15):5786. doi: 10.3390/molecules28155786 (PMC10421306; doi:10.3390/molecules28155786)
Supplement: Supplementary file 1 [file molecules-28-05786-s001.zip › Supplementary Table S3. Chromat parameters, LC-MS.pdf]

Supplementary Table S3. HPLC-DAD and LC-MS parameters used for the analysis of phenolic-rich extracts of *Asparagus* shoots

			LC-MS parameters			HPLC-DAD parameters				
Nº	Rt (min)	Phenolic compound	m/z precursor ion[a]	m/z fragment ion[b]	Ionization mode	Detection wavelength (nm)	Correlation coefficients (R^2)	LOD (µg/mL)	LOQ (µg/mL)	Recovery rate (%)
			Compounds identified by Rt and m/z ions				-	-		-
1	72.82	Isorhamnetin-3-O-glucoside (Asterin)	479,11841	-	Positive	254	-	-	-	-
2	74.20	Quercetin-3-O-rutinoside (Rutin)	609,14611	301,03474	Negative	254	0.999	0.20	0.50	98.90
3	75.69	Kaempferol-3-O-rutinoside (Nicotiflorin)	593,15119	285,03973	Negative	254	-	-	-	-
4	77.28	Isorhamnetin-3-O-rutinoside (Narcissin)	623,16176	315,05081	Negative	254	-	-	-	-
5	79.40	Quercetin	301,03538	271,02426	Negative	254	0.997	0.40	0.60	99.20
			Compounds identified by m/z ions							
6		Chelidonic acid	182,99351	139,00370	Negative	-	-	-	-	-
7		Caffeic acid	179,03498	135,04429	Negative	-	-	-	-	-
8		*Trans*-ferulic acid	193,05063	134,03643	Negative	-	-	-	-	-
9		Syringic acid	197,04555	123,00734	Negative	-	-	-	-	-
10		Sinapic acid	223,06120	121,02821	Negative	-	-	-	-	-
11		Naringenin	271,06120	153,01779	Negative	-	-	-	-	-
12		(-)-Gallocatechin	305,06668	139,03884	Negative	-	-	-	-	-
13		Quercetin 3'-methyl ether (Isorhamnetin)	315,05103	-	Negative	-	-	-	-	-
14		Myricetin	317,06558	-	Positive	-	-	-	-	-
15		*p-c*oumaric acid hexoside	325,09289	163,11152	Negative	-	-	-	-	-
16		5-*O*-*p*-coumaroylquinic acid	337,09179	271,02426	Positive	-	-	-	-	-
17		Chlorogenic acid	353,08781	163,03566	Negative	-	-	-	-	-
18		(-)-Epicatechin gallate	441,08272	-	Negative	-	-	-	-	-
19		Kaempferol-3-*O*-glucoside (Astragalin)	447,09328	-	Negative	-	-	-	-	-
20		Quercetin-3-*O*-glucoside (Isoquercetin)	463,08821	-	Negative	-	-	-	-	-
21		Pelargonidin 3-*O*-diglucoside	595,16575	271,06010	Positive	-	-	-	-	-

[a] Mass error lower than 5 ppm.
[b] Mass error lower than 10 ppm.
